# Histone deacetylase 7 mediates lipopolysaccharide-inducible mitochondrial fission in macrophages

**DOI:** 10.1242/jcs.264376

**Published:** 2025-10-10

**Authors:** Rishika Abrol, Syeda Farhana Afroz, James E. B. Curson, Karoline D. Raven, Kaustav Das Gupta, Kimberley S. Gunther, Alun Jones, Robert C. Reid, Zherui Xiong, Jennifer H. Gunter, Jessica A. Engel, Christian R. Engwerda, Antje Blumenthal, David P. Fairlie, Robert G. Parton, Steven Zuryn, Ronan Kapetanovic, Divya Ramnath, Matthew J. Sweet

**Affiliations:** ^1^ Institute for Molecular Bioscience, The University of Queensland, Brisbane, QLD 4072, Australia; ^2^ Australian Infectious Diseases Research Centre, The University of Queensland, Brisbane, QLD 4072, Australia; ^3^ Division of Infectious Disease, The Lundquist Institute for Biomedical Innovation at Harbor-University of California, Los Angeles Medical Center, Torrance, CA 90502, USA; ^4^ Queensland University of Technology (QUT), Australian Prostate Cancer Research Centre - Queensland, Centre for Genomics and Personalised Health, School of Biomedical Sciences, Translational Research Institute, Brisbane, QLD 4102, Australia; ^5^ QIMR Berghofer Medical Research Institute, Brisbane, QLD 4006, Australia; ^6^ Frazer Institute, The University of Queensland, Brisbane, QLD 4102, Australia; ^7^ ARC Centre of Excellence for Innovations in Peptide and Protein Science, Institute for Molecular Bioscience, The University of Queensland, Brisbane, QLD 4072, Australia; ^8^ Centre for Microscopy and Microanalysis, The University of Queensland, Brisbane, QLD 4072, Australia; ^9^ Clem Jones Centre for Ageing Dementia Research, Queensland Brain Institute, The University of Queensland, Brisbane, QLD 4072, Australia; ^10^ INRAE, Université de Tours, Infectiologie et Santé Publique (ISP), Nouzilly 37380, France

**Keywords:** Dynamin-related protein 1, Glycolysis, Histone deacetylase, Immunometabolism, Lysine deacetylase, Macrophages, Mitochondrial dynamics, Mitochondrial fission, Post-translational modification, Toll-like receptor

## Abstract

Histone deacetylase 7 (HDAC7) drives several immunometabolism-related processes in macrophages including lipopolysaccharide (LPS)-inducible glycolysis and inflammatory mediator production. Using an advanced biotin ligase TurboID system in human macrophages, we report 104 candidate HDAC7 interaction partners that might contribute to its immunometabolic functions. One such protein is the mitochondrial fission-promoting GTPase dynamin-related protein 1 (DRP1, also known as DNM1L), which associates with HDAC7 in cells. Using gain- and loss-of-function genetic approaches, we show that HDAC7 promotes LPS-inducible mitochondrial fission in macrophages, as well as DRP1-dependent metabolic and inflammatory responses. HDAC7 enzymatic activity was dispensable for LPS-inducible fission, as previously reported for LPS-inducible glycolysis. However, a pharmacological inhibitor of HDAC7 attenuated fission in primary human and mouse macrophages, implicating its acetyl-lysine docking function in this response. HDAC7 thus drives inducible mitochondrial fission in macrophages. Small molecules targeting the acetyl-lysine docking function of HDAC7 might have applications in preventing pathological processes driven by dysregulated mitochondrial fission.

## Teaser

**Summary:** The macrophage HDAC7 interactome includes DRP1, leading to the discovery that HDAC7 promotes LPS-inducible mitochondrial fission, as well as DRP1-dependent glycolysis and inflammatory responses.

## INTRODUCTION

Macrophages detect danger through pattern recognition receptors (PRRs) expressed on the cell surface, on endosomal compartments and within the cytosol (Takeuchi and Akira, 2010). Toll-like receptors (TLRs) are among the most widely studied PRRs, sensing and responding to threats by initiating inflammatory and antimicrobial responses, priming adaptive immunity, and controlling cell survival (Fitzgerald and Kagan, 2020). TLR signalling also reprograms macrophage metabolism, with this required for specific functional responses (O'Neill and Pearce, 2016; Kolliniati et al., 2022). For example, lipopolysaccharide (LPS)-mediated TLR4 activation increases glycolysis and signals the glycolytic enzyme pyruvate kinase isoform M2 (PKM2) to drive inflammatory gene expression via the transcription factor HIF-1α in macrophages (Palsson-McDermott et al., 2015).

Many metabolic enzymes, including PKM2, are regulated by lysine acetylation (Shakespear et al., 2018). Classical histone deacetylases (named HDAC1 to HDAC11) deacetylate target proteins and/or act as scaffolding proteins to control immune cell functions (Shakespear et al., 2011). Class IIa HDACs (HDAC4, HDAC5, HDAC7 and HDAC9) are generally considered to be enzymatically inactive because they lack a critical active-site tyrosine residue that is conserved in other classical HDAC enzymes (Lahm et al., 2007). Nonetheless, enzyme activity of one class IIa HDAC, HDAC7, is regulated in activated macrophages. Signalling via multiple TLRs rapidly increases HDAC7 enzymatic activity in macrophages, as measured by deacetylation of an artificial class IIa HDAC-specific trifluoroacetyl-lysine-containing substrate (Ramnath et al., 2022). Except for TLR3, which signals via TRIF (also known as TICAM1; Kawasaki and Kawai, 2014), TLR-mediated activation of HDAC7 enzyme activity requires the MyD88 adaptor (Ramnath et al., 2022). Upon TLR activation, HDAC7 drives PKM2-dependent activation of HIF-1α for inducible IL-1β production in macrophages (Das Gupta et al., 2020) and also promotes TLR-inducible glycolysis (Ramnath et al., 2022; Das Gupta et al., 2020), which has been linked to inflammation (Soto-Heredero et al., 2020) and chronic conditions such as rheumatoid arthritis (Umar et al., 2021). Interestingly, HDAC7 enzymatic activity is required for LPS-induced IL-1β production (Ramnath et al., 2022) but not inducible glycolysis (Ramnath et al., 2022) in macrophages, suggesting that HDAC7 has both enzyme-dependent and enzyme-independent functions in these cells. Collectively, these studies position HDAC7 as a central player in TLR-regulated immunometabolism in macrophages (Wang et al., 2023a).

Mitochondria play critical roles in macrophage functions (Angajala et al., 2018; Afroz et al., 2023). These dynamic organelles alternate between a fused mitochondrial network (fusion) and fragmented mitochondria (fission). Mitochondrial dynamics is regulated by several dynamin-related GTPases, including the fusion-promoting mitofusin 1 and 2 (Giacomello et al., 2020) and fission-promoting dynamin-related protein 1 (DRP1, also known as DNM1L), which is recruited to mitochondrial membranes (Bleazard et al., 1999) for membrane scission (Fonseca et al., 2019). Increasing evidence suggests a connection between mitochondrial fission and LPS-inducible glycolysis. LPS and other inflammatory stimuli that rewire cellular metabolism towards glycolysis (Mills et al., 2016; Jha et al., 2015) also skew mitochondrial dynamics towards fission in macrophages (Kapetanovic et al., 2020), and inhibiting mitochondrial fission reduces LPS-induced glycolytic reprogramming in various cell types (Zhang et al., 2019; Nair et al., 2019; Buck et al., 2016).

In this study, we explored mechanisms by which HDAC7 controls metabolic functions in macrophages via a proximity-based screen for HDAC7 interaction partners. Using the TurboID advanced biotin ligase system (Zhang et al., 2020) coupled with proteomic analysis, we reveal that the HDAC7 macrophage interactome includes DRP1. This led us to discover that HDAC7 promotes LPS-inducible mitochondrial fission in macrophages, as well as DRP1-dependent metabolic and inflammatory responses. Our studies also revealed a two-stage mechanism of LPS-inducible mitochondrial fission, with HDAC7 playing a particularly pronounced role in the latter. Our findings provide mechanistic insight into how TLR activation connects mitochondrial dynamics and metabolic changes in macrophages, while also revealing HDAC7 as a potential target for attenuating dysregulated mitochondrial fission in innate immune cells.

## RESULTS

### TurboID identifies 104 candidate HDAC7 interaction partners

To identify HDAC7 interaction partners, we used the TurboID advanced biotin ligase system (Branon et al., 2018). In this system, cells expressing a HDAC7–TurboID fusion protein biotinylate proximal proteins (Fig. S1A), which can then be characterised (Fig. S1B). We used a doxycycline-inducible system to conditionally express HDAC7–TurboID in THP1 cells (Stocks et al., 2021) for identifying candidate HDAC7-interacting partners. As specificity controls, we generated THP1 cells inducibly expressing TurboID alone (empty vector, EV) or natural killer granule protein 7 (NKG7)–TurboID, as an inflammation-related control protein (Ng et al., 2020). We verified doxycycline-induced expression of FLAG-tagged HDAC7 or V5-tagged NKG7 (Fig. S1C) and, using a class IIa HDAC-specific enzyme assay in which a trifluoroacetyl-lysine-containing substrate is deacetylated (Ramnath et al., 2022), we confirmed that the HDAC7–TurboID fusion protein was functional. As expected, doxycycline-stimulated HDAC7-expressing THP1 cells had elevated basal and LPS-inducible class IIa HDAC activity, compared to control cells (Fig. S1D,E).

**Fig. 1.**
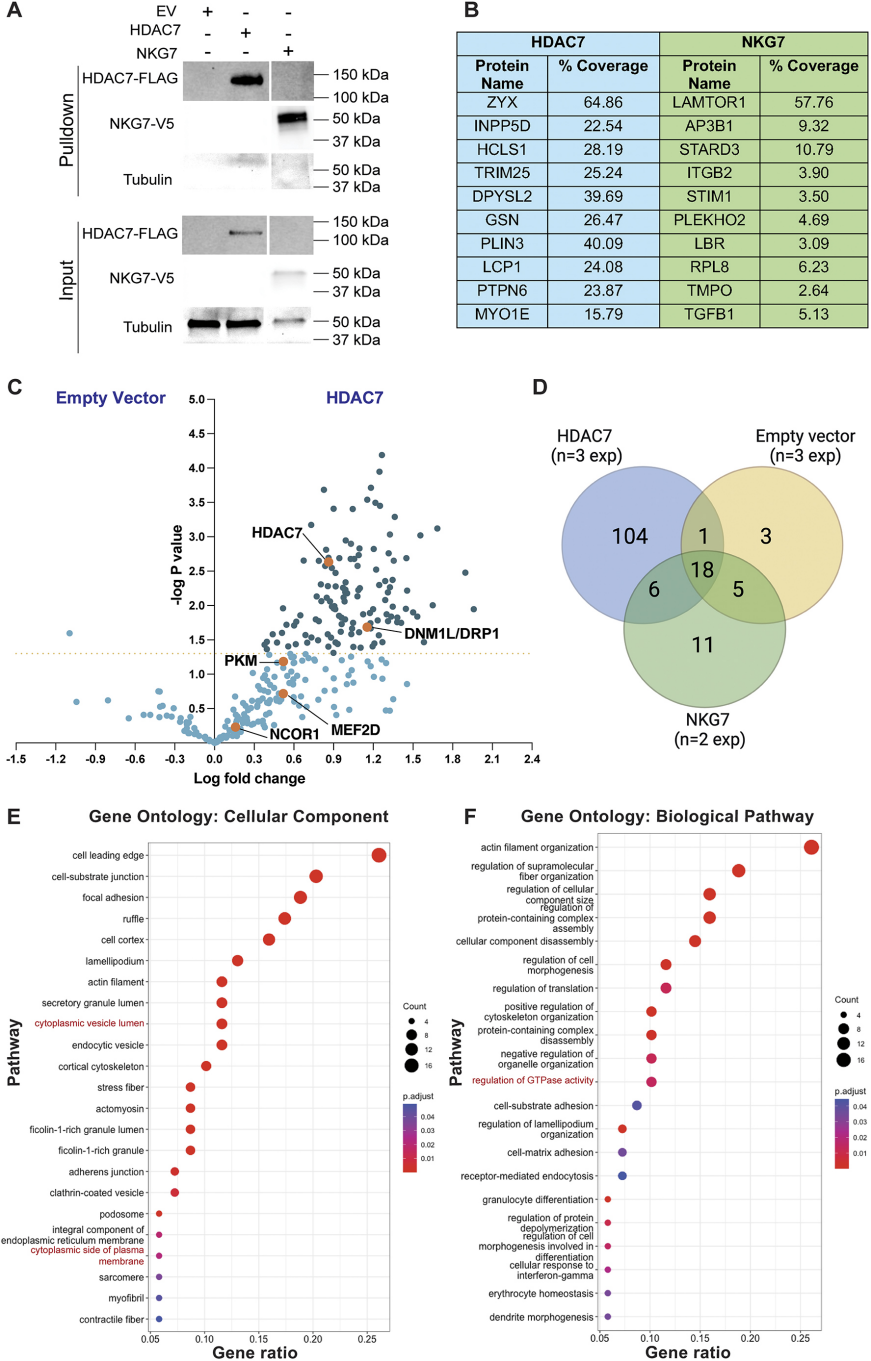
**TurboID tagging identifies 104 candidate HDAC7 interaction partners.** PMA-differentiated THP1 cell lines expressing HDAC7–FLAG–TurboID, NKG7–V5–TurboID or TurboID alone (EV) were treated with or without doxycycline, lysed, and biotinylated proteins were isolated using streptavidin pulldown. The samples were then digested using trypsin, and proteins identified by mass spectrometry. (A) Proteins in input and pulldown samples were detected by immunoblotting with anti-FLAG, anti-V5 or anti-tubulin antibodies, as indicated (*n*=3). Input shows 5% of total lysate. (B) Table shows proteins with the highest peptide coverage identified in the HDAC7–TurboID and NKG7–TurboID samples (ProteinPilot, 1% global FDR). (C) Scatter dot plot comparing mass spectrometry peak area of proteins identified in empty vector TurboID and HDAC7–TurboID (HDAC7) samples (using Skyline). The *x*-axis indicates the fold change across the two conditions, and the *y*-axis indicates statistical significance for differentially expressed proteins (dark blue dots, proteins with *P*<0.05; orange dots, proteins of interest). Statistical analysis was performed using an unpaired two-tailed *t*-test (*n*=3). (D) Venn diagram representing numbers of proteins identified by mass spectrometry across all experiments, as determined using ProteinPilot (104 proteins unique to HDAC7 in each of three experiments). (E,F) The 104 HDAC7–TurboID-specific proteins were subjected to GO (E) cellular component and (F) biological pathway enrichment analysis (using ClusterProfiler). Dot plots represent pathways enriched in proteins identified from cells expressing HDAC7–TurboID. Dot sizes indicate the protein count and colours indicate *P*-values (*n*=3). See also Fig. S1.

To identify candidate HDAC7 interaction partners, we isolated biotinylated proteins by streptavidin pulldown in THP1 cells expressing TurboID fusion proteins (Xiong et al., 2021). As expected, the HDAC7 and NKG7 bait fusion proteins were themselves biotinylated, as indicated by their detection in the streptavidin pulldown samples (Fig. 1A). Other biotinylated proteins were identified by mass spectrometry, with the top ten proteins specific to HDAC7 and NKG7 displayed in Fig. 1B, and proteins significantly enriched in the HDAC7–TurboID versus EV–TuboID pulldowns shown in Fig. 1C (124 proteins were significantly enriched in the HDAC7–TurboID samples, with at least two peptides detected at greater than 95% confidence). Known HDAC7 interaction partners, such as PKM2 (Das Gupta et al., 2020), 14-3-3 proteins (Li et al., 2004), NCOR1 (Fischle et al., 2001) and MEF2D (Aude-Garcia et al., 2010) were all unique to the HDAC7–TurboID pulldowns (Fig. 1C), thus validating our approach. After false discovery rate correction, 104 proteins were unique to the HDAC7–TurboID pulldown samples when compared to the EV and NKG7–TurboID controls in each of three independent experiments (Fig. 1D; Table S1). To gain insights into the HDAC7 interactome, Gene Ontology (GO) and KEGG pathway analyses were employed (Chen et al., 2017) (Fig. 1E,F; Fig. S1F,G). The set of HDAC7 partner proteins was significantly enriched with proteins associated with cytoplasmic compartments (Fig. 1E), where HDAC7 is localised in macrophages (Ramnath et al., 2022). GO analysis also revealed that this set of proteins was enriched with proteins involved in several specific pathways, including actin filament organisation and regulation of GTPase activity (Fig. 1F; Fig. S1F). KEGG analysis also revealed the involvement of HDAC7 partners in bacterial infection and phagocytosis (Fig. S1G), consistent with known HDAC7 functions (Das Gupta et al., 2023). These findings broadly validate the candidate HDAC7 interaction partners identified.

### HDAC7 associates with the mitochondrial fission protein DRP1

**Fig. 2.**
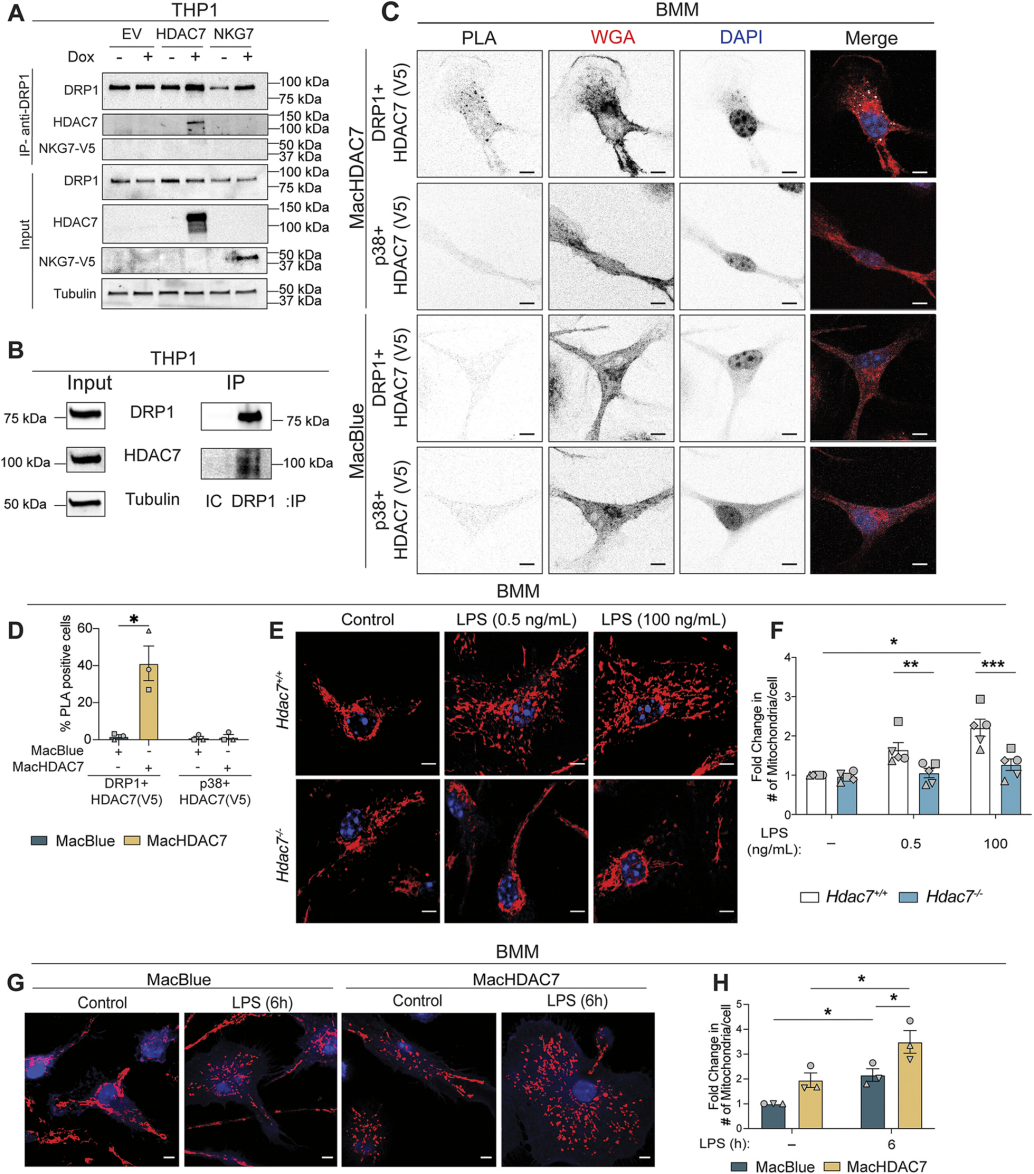
**HDAC7 associates with mitochondrial fission protein DRP1 and drives LPS-inducible fission in macrophages.** (A) Indicated PMA-differentiated THP1 stable cell lines were treated with or without doxycycline (Dox) then lysed, and proteins were immunoprecipitated (IP) with an anti-DRP1 antibody. Immunoblotting was used to visualise HDAC7 (anti-FLAG antibody), DRP1 (anti-DRP1 antibody), NKG7 (anti-V5 antibody) and tubulin (anti-tubulin antibody) (*n*=3). Input shows 25% of total lysate. (B) PMA-differentiated THP1 cells were crosslinked using formaldehyde then lysed, after which proteins were immunoprecipitated with an anti-DRP1 antibody or an isotype control (IC). Immunoblotting was used to visualise DRP1, HDAC7 and tubulin in inputs and immunoprecipitates (*n*=2). Input shows 10% of total lysate. (C,D) MacHDAC7 and MacBlue BMMs were fixed, and a PLA was performed using antibodies against the V5 tag (HDAC7–V5) and either endogenous DRP1 or MAPK p38 (control). (C) Cells were stained with DAPI (blue) and Alexa Fluor 647-conjugated wheat germ agglutinin (WGA; red) then visualised by confocal microscopy (*n*=3). Scale bars: 5 µm. (D) The percentage of PLA-positive cells in each condition was quantified using a threshold of ≥3 puncta/cell. Data are shown as mean±s.e.m., *n*=3. *P<0.05 (repeated measures two-way ANOVA with Šidák's multiple comparisons test). (E) *Hdac7^+/+^* and *Hdac7^−/−^* BMMs were stimulated with LPS (0.5 ng/ml, 100 ng/ml) for 6 h, stained with MitoTracker (red) and DAPI (blue), then visualised by confocal microscopy (*n*=5). Scale bars: 5 µm. (F) Mitochondrial numbers per cell from images in E were quantified. Data are shown as mean±s.e.m., normalised to unstimulated *Hdac7^+/+^* controls (*n*=5). *P<0.05; **P<0.01; ***P<0.001 (repeated measures two-way ANOVA with Dunnett's multiple comparisons test). (G) MacBlue and MacHDAC7 BMMs were stimulated with LPS (100 ng/ml) for 6 h. Cells were stained with MitoTracker (red) and DAPI (blue), then visualised by confocal microscopy (*n*=3). Scale bars: 5 µm. (H) Mitochondrial numbers per cell from images in G were quantified. Data are shown as mean±s.e.m., normalised to unstimulated MacBlue controls (*n*=3). *P<0.05 (repeated measures two-way ANOVA with Tukey's multiple comparisons test). See also Fig. S2.

We focused on HDAC7-specific candidates that might contribute to HDAC7-dependent TLR-inducible glycolysis (Das Gupta et al., 2020). Five glycolysis-related proteins [PKM, G6PD, DRP1 (DNM1L), HSPA5 and GNB2L1 (RACK1)] (Zhang et al., 2019; Liberzon et al., 2015; Xu et al., 2022) were identified (Fig. S1H). DRP1 is essential for mitochondrial fission (Kapetanovic et al., 2020) and glycolysis (Zhang et al., 2019; Na et al., 2023; Seo et al., 2020) in various cell types, so was selected for further analysis. We next employed additional methods to assess the association between HDAC7 and DRP1 in cells*.* Immunoprecipitation of DRP1 with an antibody against endogenous DRP1 also pulled down ectopically expressed HDAC7 in stably transduced THP1 cells (Fig. 2A). Using co-immunoprecipitation, we additionally confirmed an association between endogenous HDAC7 and DRP1 in phorbol 12-myristate 13-acetate (PMA)-differentiated THP1 cells (Fig. 2B). To further validate this interaction, proximity ligation assays (PLAs) were performed on bone marrow-derived macrophages (BMMs) from either mice overexpressing V5-tagged HDAC7 in myeloid cells (MacBlue×UAS-HDAC7 mice, hereafter MacHDAC7 mice) (Das Gupta et al., 2020) or littermate control MacBlue mice. In this system, a tissue-specific promoter restricts expression of the GAL4-VP16 transcriptional activator to myeloid cells in MacBlue mice. Consequently, when MacBlue mice are crossed with UAS-HDAC7 mice, mice that inherit both transgenes (MacHDAC7 mice) express high levels of HDAC7–V5 in macrophages (Das Gupta et al., 2020). These PLA experiments with anti-V5 and anti-DRP1 antibodies revealed that HDAC7 and DRP1 are in close proximity in primary macrophages from MacHDAC7 mice, with a PLA signal not detected using the same antibody pair in MacBlue control BMMs (Fig. 2C,D). The specificity of the interaction was also confirmed using antibodies against HDAC7–V5 and p38 mitogen-activated protein kinase (MAPK) as an unrelated cytoplasmic control protein.

### HDAC7 is essential for LPS-inducible mitochondrial fission in macrophages

We previously showed that LPS promotes DRP1-dependent mitochondrial fission in macrophages, without affecting mitochondrial mass or mitochondrial DNA replication (Kapetanovic et al., 2020). To investigate the significance of the HDAC7 and DRP1 association, LPS-inducible mitochondrial fission was assessed in *Hdac7*-deficient BMMs. Stimulation with sub-maximal (0.5 ng/ml) or maximal stimulatory (100 ng/ml) LPS concentrations for 6 h led to mitochondrial fragmentation and an increase in mitochondria numbers in *Hdac7^+/+^* control BMMs (Fig. 2E,F; Fig. S2A). This LPS-inducible increase in mitochondrial numbers is dependent on DRP1 in macrophages, indicating increased mitochondrial fission (Kapetanovic et al., 2020). Consistent with this, LPS treatment reduced mitochondrial branch length, a measure of the average length of connected mitochondrial segments (Fig. S2B), as well as form factor, a composite measure of mitochondrial shape complexity and elongation (Fig. S2C). Reductions in these parameters indicate a shift toward a more fragmented, punctate mitochondrial network. In contrast, LPS did not promote mitochondrial fission in *Hdac7*-deficient macrophages (Fig. 2E,F; Fig. S2A–C). This defect could not be explained by perturbations in mitochondrial mass, which was unaffected by *Hdac7* deficiency in BMMs (Fig. S2D). Furthermore, the failure of LPS to induce fission in *Hdac7*-deficient BMMs was not reflective of a broader defect in LPS responsiveness, as these cells retained the ability to respond to LPS with inducible expression of *Tnf* mRNA (Fig. S2E) (Das Gupta et al., 2020). We next utilised gain-of-function MacHDAC7 mice (Das Gupta et al., 2020) that overexpress HDAC7 in macrophages to further test the role of HDAC7 in fission. MacHDAC7 BMMs displayed increased basal and LPS-inducible mitochondrial fission by comparison to MacBlue control BMMs, with the inducible response being enhanced at both low dose (0.5 ng/ml) and high dose (100 ng/ml) LPS concentrations (Fig. 2G,H; Fig. S2F,G). Collectively, these data indicate that HDAC7 mediates LPS-induced fission in macrophages at 6 h post-stimulation.

### HDAC7 enzyme activity is dispensable for LPS-inducible fission

**Fig. 3.**
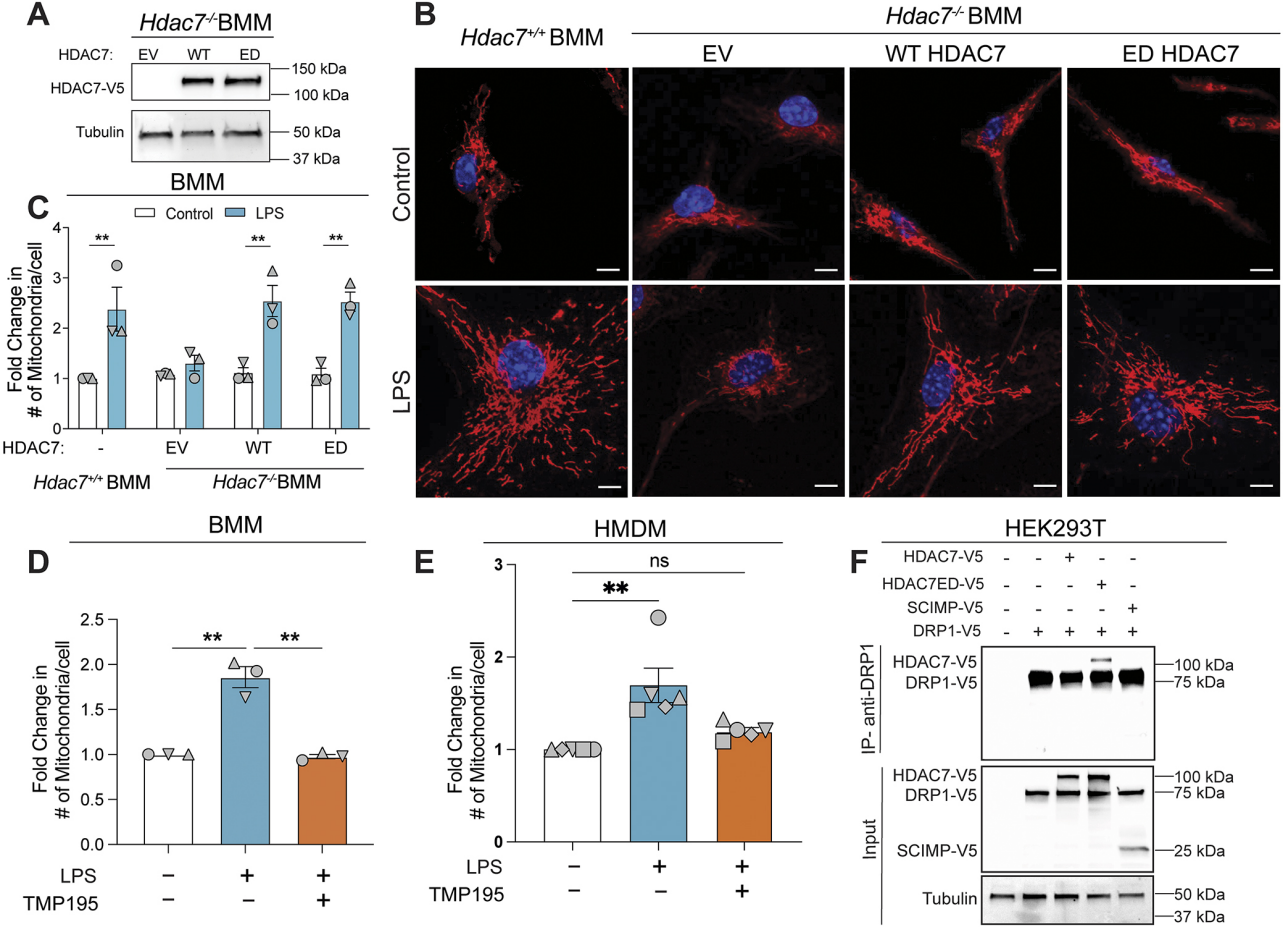
**HDAC7 enzyme activity is dispensable for LPS-inducible fission.** (A–C) *Hdac7^−/−^* BMMs were transduced with empty vector (EV), WT HDAC7 or ED HDAC7, with *Hdac7^+/+^* BMMs used as a positive control. (A) HDAC7 protein expression in transduced *Hdac7^−/−^* BMMs (anti-V5 antibody). *n*=3. (B) The indicated cell lines were stimulated with LPS (100 ng/ml) for 6 h, stained with MitoTracker (red) and DAPI (blue), then visualised by confocal microscopy. Scale bars: 5 µm. (C) Mitochondrial numbers per cell from images in B were quantified Data are shown as mean±s.e.m., normalised to WT BMM unstimulated control (*n*=3). **P<0.01 (repeated measures two-way ANOVA with Šidák's multiple comparisons test). (D) BMMs were pre-treated with DMSO or TMP195 (0.5 μM) for 1 h, then stimulated with LPS (100 ng/ml) for 6 h, as indicated. Cells were stained with MitoTracker and DAPI, visualised by confocal microscopy, and mitochondrial numbers per cell were quantified. Data are shown as mean±s.e.m., normalised to the unstimulated DMSO-treated control (*n*=3). **P<0.01 (repeated measures one-way ANOVA with Dunnett's multiple comparisons test). (E) HMDMs were pre-treated with DMSO or TMP195 (10 μM) for 1 h, then stimulated with LPS (100 ng/ml) for 6 h. Cells were stained with MitoTracker and DAPI, visualised by confocal microscopy, and mitochondrial numbers per cell were quantified. Data are shown as mean±s.e.m., normalised to the unstimulated DMSO-treated control (*n*=5). **P<0.01; ns, not significant (repeated measures one-way ANOVA with Dunn's multiple comparisons test). (F) HEK293T cells were co-transfected with V5-tagged DRP1 and either HDAC7, enzyme-dead HDAC7 (HDAC7ED) or SCIMP (unrelated control), as indicated. Cells were lysed, samples were immunoprecipitated (IP; using anti-endogenous DRP1), and immunoblotting was used to visualise the V5 tag (detecting DRP1 and candidate interactors) and tubulin (*n*=3). Input shows 25% of total lysate. See also Figs S3, S4, S5 and S6.

Acute LPS-inducible glycolysis in macrophages does not require HDAC7 enzyme activity (Ramnath et al., 2022). We therefore predicted that HDAC7 enzyme activity would be dispensable for LPS-inducible mitochondrial fission. To examine this, we used an HDAC7 point mutant that lacks catalytic activity due to the mutation of a key histidine residue to alanine in the active site (H649A), as previously described (Das Gupta et al., 2020). Hereafter, we refer to this mutant as enzyme-dead (ED) HDAC7. Reconstitution of *Hdac7*-deficient BMMs with wild-type (WT) or ED HDAC7 (Fig. 3A) (Das Gupta et al., 2020), but not the empty vector control (EV), rescued LPS-inducible mitochondrial fission (Fig. 3B,C; Fig. S3A) without affecting DRP1 protein levels (Fig. S3B). Interestingly however, TMP195, a pharmacological inhibitor of class IIa HDACs (Guerriero et al., 2017), attenuated LPS-inducible fission in mouse BMMs (Fig. 3D; Fig. S3C) and human monocyte-derived macrophages (HMDMs) (Fig. 3E; Fig. S3D). This further supports a requirement for HDAC7 in LPS-inducible fission in macrophages and implies that the TMP195-binding pocket might be required. We further investigated this by examining the interaction between DRP1 and HDAC7-WT versus HDAC7-ED in immunoprecipitation experiments in co-transfected HEK293T cells, with TLR signalling protein SCIMP (Luo et al., 2017) serving as a negative control (Fig. 3F). Whereas DRP1 associated with WT HDAC7 in macrophages (Fig. 2A–D), it immunoprecipitated HDAC7-ED but not HDAC7-WT in the HEK293T cell co-transfection system. The reasons for this are unclear but might relate to cell type-specific differences and/or different sensitivities in the methods used. Since HDAC7 enzyme activity was not required for LPS-induced fission (Fig. 3B,C), this enhanced interaction with ED HDAC7 is unlikely due to increased binding of DRP1 as a substrate. Instead, we presume the enzymatic pocket of HDAC7 facilitates the interaction between HDAC7 and DRP1, with this pocket being more accessible in the ED form of HDAC7. Interestingly, DRP1 also interacted with class IIa HDAC5 and class IIb HDAC6 in co-transfected HEK293T cells (Fig. S3E). This suggests that additional factors beyond DRP1 binding alone are required for the non-redundant role of HDAC7 in driving LPS-inducible mitochondrial fission in macrophages (Fig. 2E,F). Collectively, our data suggest that the acetyl-lysine docking function of HDAC7, but not its enzymatic activity, is likely to mediate LPS-inducible mitochondrial fission.

### HDAC7 has different roles in transient versus sustained LPS-inducible mitochondrial fission

To gain further insight into HDAC7-dependent mitochondrial fission, we considered the kinetics of the response. We previously provided evidence of two distinct stages of LPS-induced mitochondrial fission, with LPS triggering transient DRP1 phosphorylation at S635 (numbering based on NP_001346936.1; corresponds to human S616), followed by subsequent DRP1 dephosphorylation at S656 (corresponds to human S637) at 6 h post-LPS stimulation and beyond (Kapetanovic et al., 2020). To test the involvement of these post-translational modifications (PTMs), we reconstituted *Drp1*-deficient MEF cells (Kapetanovic et al., 2020) with a WT DRP1 construct, a phosphodeficient S635A DRP1 mutant (predicted to be defective in early stage fission) and a phosphomimetic S656E DRP1 mutant (predicted to be defective in late stage fission). As expected, reconstitution of WT DRP1 in *Drp1^−/−^* MEF cells restored the early (3 h) and late (8 h) LPS-inducible fission responses, with effects being comparable to WT MEF cells (Fig. S4A,B). Moreover, DRP1-S635A-expressing MEF cells were defective in the first but not the second stage of inducible fission, whereas the reverse was true for DRP1-S656E-expressing MEF cells. These data support a model in which LPS-induced DRP1 S635 phosphorylation permits a first stage of fission, with subsequent S656 dephosphorylation enabling a second stage of fission.

Next, we used *Hdac7*-deficient BMMs to examine the role of HDAC7 in these two stages of fission. HDAC7 was only partially required for inducible fission at 2 h post-LPS stimulation, whereas the response was completely HDAC7-dependent by 6 h (Fig. S5A,B). These defects were not due to reduced DRP1 protein levels or overall mitochondrial content as assessed by TOM20 (TOMM20) protein levels, both of which were similar between WT and *Hdac7*-deficient BMMs over an LPS time course (Fig. S5C–E). Assessing DRP1 modifications, HDAC7 was not required for inducible DRP1-S635 phosphorylation (Fig. S5C,F), despite it having a partial involvement in the first stage of LPS-induced fission (Fig. S5A,B). In contrast, the apparent DRP1-S656 dephosphorylation, as detected with a phospho-DRP1 (pDRP1) S656 antibody (Cell Signaling Technology, #4867S), was abrogated in *Hdac7*^−/−^ BMMs (Fig. S5C,G). This antibody detected a higher molecular mass band of greater than 90 kDa (instead of ∼80 kDa for DRP1), which we presumed to be a SUMOylated version of DRP1 corresponding to this size (Figueroa-Romero et al., 2009; Prudent et al., 2015). However, this band was still detected in *Drp1*^−/−^ MEF cells when using the pDRP1 S656 antibody, whereas this was not the case for an antibody detecting total DRP1 (Fig. S5H). This suggests that the pDRP1 S656 antibody (CST, #4867S) detects a cross-reactive protein but not DRP1 itself (hereafter referred to as fission-related protein, FRP). Given that DRP1-S656 dephosphorylation was required for the second stage of fission (Fig. S4A,B) and that HDAC7 was required for both the second stage of LPS-induced fission (Fig. S5A,B) and the corresponding loss of FRP (Fig. S5C,G), we conclude that the LPS-induced decrease in FRP correlates with DRP1-S656 dephosphorylation.

To further probe how HDAC7 might connect to the second wave of LPS-inducible fission, we explored other signalling components. As expected, TLR4 was required for both the first and second stages of inducible fission (Fig. S6A), as well as acute DRP1-S635 phosphorylation and the subsequent loss of FRP (Fig. S6B). We next assessed MyD88 involvement, since this adaptor is required for inducible fission at 6 h post-LPS stimulation (Kapetanovic et al., 2020). Here we observed that acute LPS-inducible fission did not require MyD88 (Fig. S6C,D). This presumably reflects the involvement of other TLR adaptors, since TAK-242 (resatorvid), which antagonises MAL (TIRAP)- and TRAM-mediated signalling (Matsunaga et al., 2011; Kawamoto et al., 2008), attenuated both stages of inducible fission (Fig. S6E) and acute DRP1-S635 phosphorylation (Fig. S6F). We conclude that HDAC7 partially contributes to the first stage of TLR4-induced fission independently of DRP1-S635 phosphorylation, whereas it is essential for the subsequent sustained second stage of fission, likely by connecting MyD88 to DRP1-S656 dephosphorylation.

### HDAC7 promotes LPS-inducible glycolysis and a subset of inflammatory responses via DRP1

**Fig. 4.**
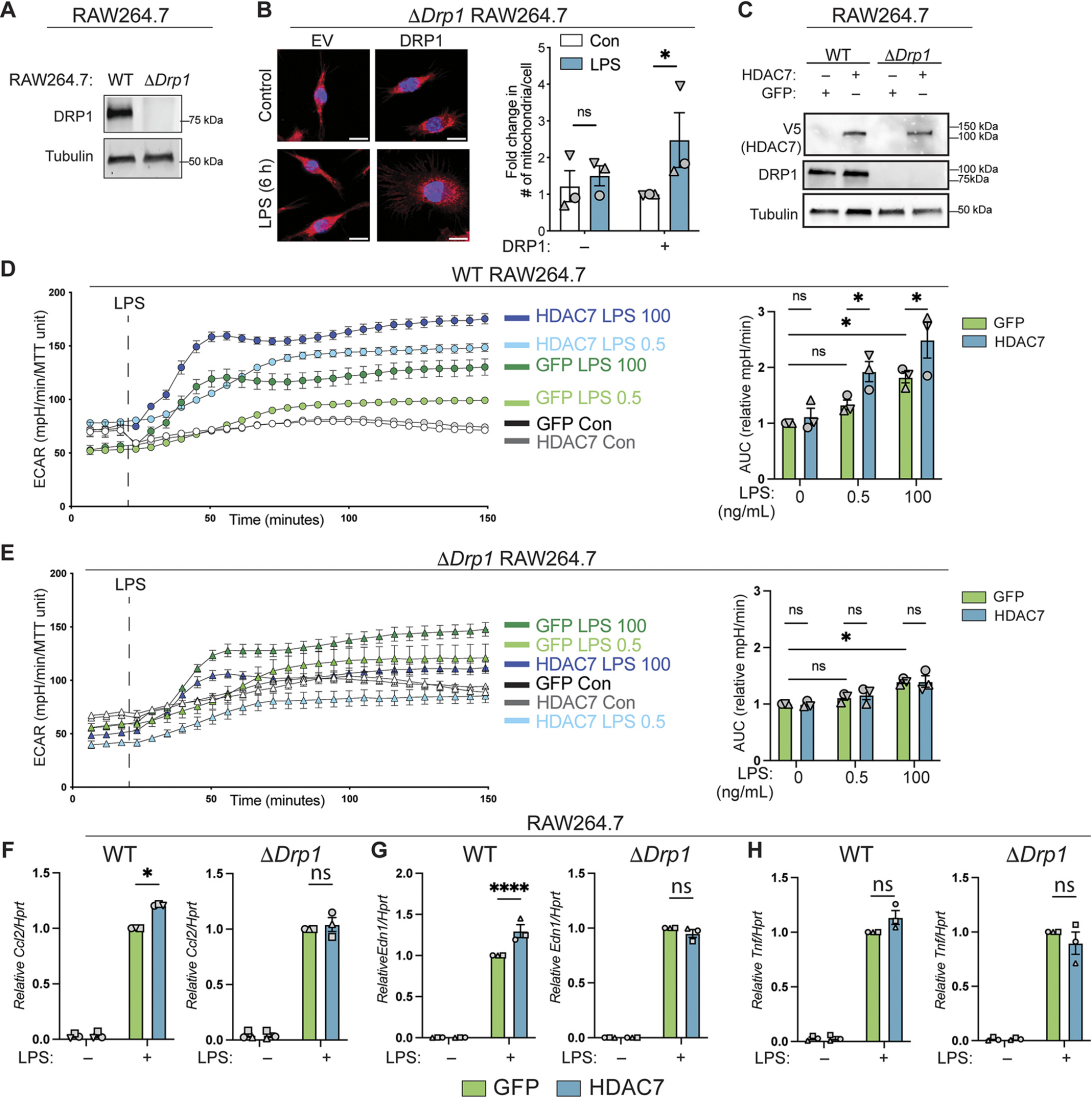
**HDAC7 requires DRP1 to promote LPS-inducible glycolysis and to fine-tune inflammatory gene expression in macrophages.** (A) Immunoblotting was used to assess DRP1 protein levels in WT and *Drp1*-deficient (Δ*Drp1*) RAW264.7 cells generated using CRISPR-Cas9. *n*=3. (B) Left: Δ*Drp1* RAW264.7 cells were transfected with empty vector (EV) or DRP1 plasmids, stimulated with LPS (100 ng/ml) for 6 h, stained with MitoTracker (red) and DAPI (blue), then visualised by confocal microscopy. Scale bars: 10 µm. Right: mitochondrial numbers per cell were quantified. Data are shown as mean±s.e.m., normalised to the DRP1-transfected unstimulated control (*n*=3). *P<0.05; ns, not significant (repeated measures two-way ANOVA with Šidák's multiple comparisons test). (C) WT and Δ*Drp1* RAW264.7 cells were transfected with mRNAs encoding either HDAC7 or GFP (control), as indicated, after which levels of HDAC7 (anti-V5 antibody), DRP1 and tubulin were assessed by immunoblotting. *n*=3. (D) WT and (E) Δ*Drp1* RAW264.7 cells were transfected with mRNAs encoding either HDAC7 or GFP, after which ECAR levels were measured following treatment with LPS or in untreated controls (Con), as indicated. Left: ECAR plots from a representative experiment. Data plotted are mean±s.e.m. from *n*=3 (experimental triplicates). Right: area under the curve (AUC) analysis. Data are shown as mean±s.e.m., normalised to the LPS-treated GFP control (*n*=3). *P<0.05; ns, not significant (two-way ANOVA with Šidák's post test). (F–H) WT and Δ*Drp1* RAW264.7 cells were transfected with mRNAs encoding either HDAC7 or GFP (control). After 24 h, cells were stimulated with LPS (100 ng/ml) for 4 h, as indicated, after which mRNA levels of (F) *Ccl2*, (G) *Edn1* and (H) *Tnf* (relative to *Hprt*) were quantified by qPCR. Data are shown as mean±s.e.m., normalised to the LPS-treated GFP control (*n*=3). *P<0.05; ****P<0.0001; ns, not significant (two-way ANOVA with Šidák's post test).

To investigate the potential biological significance of HDAC7-dependent TLR-inducible fission, we examined the effect of HDAC7 overexpression on metabolic and inflammatory responses in macrophages lacking DRP1. To do so, we generated *Drp1*-deficient RAW264.7 cells using CRISPR (Fig. 4A) and confirmed that DRP1 reconstitution restored inducible fission in these cells (Fig. 4B). To determine whether HDAC7 promotes LPS-inducible glycolysis via DRP1, we transfected parent and *Drp1*-deficient RAW264.7 cells with mRNAs encoding HDAC7 or GFP as a control (Fig. 4C), then measured extracellular acidification rate (ECAR) as a proxy for glycolytic activity (Fig. 4D,E). We noted that unstimulated *Drp1*-deficient RAW264.7 cells exhibited a higher ECAR baseline than parental RAW264.7 cells, highlighting metabolic heterogeneity between clonal populations of this cell line. For this reason, we focused on the effects of HDAC7 on metabolic and inflammatory responses in each cell population individually. In parental RAW264.7 cells, LPS increased the ECAR in a dose-dependent manner, and this effect was further enhanced by HDAC7 overexpression (Fig. 4D, right). In contrast, LPS only modestly increased the ECAR in *Drp1*-deficient RAW264.7 cells, and HDAC7 overexpression had no additional effect (Fig. 4E, right). Thus, DRP1 is required for HDAC7 to promote LPS-inducible glycolysis in macrophages.

Finally, we assessed whether HDAC7 enhances inflammatory gene expression via DRP1 in macrophages. Here we found that ectopic HDAC7 expression in parental but not *Drp1*-deficient RAW264.7 cells modestly but significantly increased LPS-inducible mRNA levels of two previously reported HDAC7 inflammatory gene targets, *Ccl2* (Das Gupta et al., 2020) and *Edn1* (Shakespear et al., 2013), which encode the monocyte-recruiting chemokine CCL2 and the potent vasoconstrictive and inflammatory peptide endothelin-1, respectively (Fig. 4F,G). These effects were selective, as LPS-inducible *Tnf* mRNA levels were not affected by HDAC7 in either cell line (Fig. 4H). Collectively, these data indicate that HDAC7 requires DRP1 to both promote LPS-inducible glycolysis and to fine-tune inflammatory responses in macrophages.

## DISCUSSION

HDAC7 has key roles in regulating metabolic, inflammatory and antimicrobial responses in macrophages (Ramnath et al., 2022; Das Gupta et al., 2020, 2023; Shakespear et al., 2013). In this study, we provide mechanistic insights into immunometabolic functions of HDAC7 in these cells. We show that this class IIa HDAC interacts with the GTPase DRP1, that it is essential for LPS-inducible mitochondrial fission, and that it promotes LPS-inducible glycolysis and fine-tunes inflammatory responses via DRP1. As far as we are aware, this is the first published evidence linking HDAC7 to DRP1 functions and/or mitochondrial dynamics in any biological system. There are, however, a small number of studies implicating HDAC7 in mitochondrial biology. For example, HDAC7 has been found to localise to mitochondria in prostate epithelial cells and to redistribute to the cytoplasm during cellular apoptosis (Bakin and Jung, 2004). HDAC7 has also been shown to negatively regulate genes encoding TCA cycle enzymes in the context of renal cell carcinoma (Nam et al., 2021), but this is not related to direct effects on mitochondria. In contrast, we introduce HDAC7 as a direct regulator of mitochondrial dynamics in macrophages.

Mitochondrial fission is generally indicative of cells being skewed towards glycolysis (Buck et al., 2016), with evidence supporting a functional connection between mitochondrial fission and glycolysis. LPS promotes mitochondrial fission in several cell types (Kapetanovic et al., 2020; Park et al., 2015; Katoh et al., 2017), with inhibition of fission reducing glycolytic reprogramming (Nair et al., 2019; Buck et al., 2016). In T cells, glycolytic gene expression and glycolysis require DRP1, with *Drp1*-deficient T cells being skewed towards oxidative phosphorylation (Simula et al., 2018). Similarly, DRP1 has been found to be required for the metabolic shift towards glycolysis during somatic cell reprogramming (Cha et al., 2021). Given these functional connections between mitochondrial fission and metabolic reprogramming, it seems likely that HDAC7 promotes LPS-inducible glycolysis in macrophages (Ramnath et al., 2022; Das Gupta et al., 2020), at least in part, via inducible mitochondrial fission. This is consistent with our observations that HDAC7 enzyme activity was not required for either LPS-inducible mitochondrial fission (Fig. 3B,C) or acute inducible glycolysis (Ramnath et al., 2022) in macrophages, and that HDAC7 failed to enhance LPS-inducible glycolysis in *Drp1*-deficient macrophages (Fig. 4D,E).

Since HDAC7 enzyme activity was not required for LPS-inducible fission, HDAC7 probably mediates these effects independently of protein deacetylation. Indeed, a conserved active site tyrosine present in most classical HDACs is replaced with a histidine in HDAC7 and other class IIa HDACs, resulting in this sub-class of HDACs having very weak lysine deacetylase activity *in vitro* (Lahm et al., 2007). Although the N-terminal domain of HDAC7 interacts with a range of proteins to control cell functions (Wang et al., 2023a), it seems more likely that HDAC7 promotes LPS-inducible fission by docking an acetyl-lysine-containing protein into the C-terminal enzymatic pocket, given that TMP195 (Lobera et al., 2013) inhibited LPS-inducible fission (Fig. 3D,E). However, TMP195 inhibits all class IIa HDACs (Lobera et al., 2013), and we cannot exclude the possibility that other class IIa HDACs might also be essential for LPS-inducible fission, particularly given that multiple HDACs can interact with DRP1 in cells (Fig. S3E).

Past studies on regulated mitochondrial dynamics have primarily focused on DRP1 PTMs, including phosphorylation (Afroz et al., 2023). For example, phosphorylation of rat DRP1 at S585 (mouse S635) has been associated with DRP1 recruitment to mitochondria and mitochondrial fission (Taguchi et al., 2007). In line with the transient kinetics of LPS-inducible DRP1-S635 phosphorylation in macrophages (Kapetanovic et al., 2020), we confirmed that this PTM mediates acute LPS-induced mitochondrial fission. Furthermore, this early phosphorylation of DRP1-S635 is independent of both MyD88 (Kapetanovic et al., 2020) and HDAC7 (Fig. S5C,F). In contrast, the later dephosphorylation of DRP1 enhances DRP1 GTPase activity (Cribbs and Strack, 2007; Merrill et al., 2013). DRP1 reconstitution experiments implicate DRP1-S656 dephosphorylation in the late second stage (8 h) of LPS-induced fission (Fig. S4A,B). Both HDAC7 (Fig. S5A,B) and MyD88 (Kapetanovic et al., 2020) are required for the late fission response in macrophages, so it seems likely that a MyD88–HDAC7 axis coordinates DRP1 activation, potentially via DRP1 dephosphorylation at S656, for sustained LPS-inducible fission.

HDAC7 has been widely implicated in the development and/or pathology of certain cancers, metabolic conditions and inflammatory diseases (Wang et al., 2023a). It likely contributes to inflammation-related pathology through multiple mechanisms; for example, reinforcing Th17 cell transcriptional programs in T cells (Cheung et al., 2024) and promoting immunometabolism-related pro-inflammatory cytokines in macrophages (Das Gupta et al., 2020). The connection between HDAC7 and mitochondrial fission identified here might provide further insights into how this protein drives dysregulated inflammation. Activated microglia produce inflammatory mediators that contribute to neuron destruction in Parkinson's disease and Alzheimer's disease (Gao et al., 2023), with TLR signaling implicated in these processes (Kumar, 2019). Pharmacological and genetic approaches link excessive mitochondrial fission to inflammation (Afroz et al., 2023) and neurodegeneration (Shi et al., 2023). Although HDAC7 has neuroprotective functions in neurons (Ma and D'Mello, 2011; Wu and Li, 2022), our findings suggest that it might also drive fission-mediated pathology in activated microglia. For example, HDAC7 amplified LPS-inducible expression of genes encoding the inflammatory mediators CCL2 and endothelin-1 via DRP1 (Fig. 4F,G), and both of these mediators have been implicated in various neuroinflammatory conditions (D'Orléans-Juste et al., 2019; Singh et al., 2021). Excessive mitochondrial fission also occurs in metabolic conditions, such as type II diabetes (T2D) (Wang et al., 2023b). Elevated *HDAC7* mRNA levels, along with reduced insulin secretion, have been observed in pancreatic islet cells from people with T2D compared to non-diabetic controls (Daneshpajooh et al., 2018). A similar reduction in insulin secretion has been observed in HDAC7-overexpressing rat islet and clonal β-cells, along with a reduction in mitochondrial respiration in those cells (Daneshpajooh et al., 2017). Whether the HDAC7–DRP1–fission axis contributes to this dysregulation remains an open question.

### Study limitations and future directions

Although this study has revealed that HDAC7 interacts with DRP1 and drives TLR-inducible mitochondrial fission in macrophages, the precise mechanisms by which it controls DRP1 functions are unknown. The significance of HDAC7-mediated fission in macrophages to different pathological processes – for example, neuroinflammatory and metabolic condition – also remains to be determined. Finally, HDAC7 is localised to the cytoplasm in macrophages (Ramnath et al., 2022), and subcellular localisation of HDAC7 dictates its functions in different cell types (Wang et al., 2023a), so it is unclear whether HDAC7 promotes fission in cell types other than macrophages. Thus, future studies should determine whether HDAC7 promotes mitochondrial fission in other cell types in which this protein is cytoplasmic, the precise mechanisms by which it does so, and the contribution of HDAC7-mediated fission to physiological and pathophysiological processes.

## MATERIALS AND METHODS

### Experimental models

#### Animals

Mice aged 8–12 weeks were used for the study. WT (C57BL/6) mice were acquired from an in-house breeding colony. *Hdac7^+/+^(Hdac7^fl/fl^)* were kindly provided by Professor Eric Olson and Professor Rhonda Bassel-Duby (UT Southwestern Medical Centre, TX, USA), which were further crossed with LysM^Cre^ mice to obtain myeloid specific *Hdac7*-deficient mice (*Hdac7^fl/fl^/Lysm^Cre^*) (Das Gupta et al., 2020). MacBlue mice express cyan fluorescent protein (CFP) in monocytes and macrophages due to the presence of two transgenes including the *c-fms/Csf1r* promoter that drives expression of the transcriptional activator Gal4-VP16 and UAS-CFP (Ovchinnikov et al., 2008). MacBlue mice were then crossed with C57BL/6J expressing the UAS-HDAC7-u-V5 transgene to generate progeny that expresses HDAC7-u (Margariti et al., 2009) specifically in the myeloid compartment (MacHDAC7) (Das Gupta et al., 2020). *Tlr4*^−/−^ and *MyD88*^−/−^ mice on a C57BL/6 background were used as previously described (Kapetanovic et al., 2020).

#### Primary cell culture

To generate murine BMMs, bone marrow cells were isolated from the femurs and tibias of mice. Post-euthanasia, the bones were sterilised using 70% ethanol and then flushed using a 27-gauge needle containing PBS to isolate the cells. The obtained cells were then cultured in RPMI1640 medium (Invitrogen) with 2 mM L-glutamine (Gibco), 10% fetal bovine serum (FBS) (Gibco), 50 U/ml penicillin (Invitrogen), 50 µg/ml streptomycin (Invitrogen) and 150 ng/ml human colony stimulating factor-1 (CSF-1; Protein Expression Facility, The University of Queensland). Cells were harvested on day 6 of BMM differentiation and plated as required for experiments.

HMDMs were generated by positive selection of CD14-positive monocytes from buffy coats obtained from the Australian Red Cross Lifeblood. In brief, buffy coat samples were subjected to Ficoll (Ficoll-Paque Plus, GE Healthcare Life Sciences, USA) density gradient centrifugation to isolate peripheral blood mononuclear cells. Subsequently, the cells were incubated with CD14+ Microbeads (Miltenyi Biotech, Germany) and transferred into LS columns (Miltenyi Biotech, Germany). The eluted CD14-positive monocytes were then cultured in IMDM media (Gibco) with 10% FBS, 50 U/ml penicillin, 50 µg/ml streptomycin and 150 ng/ml CSF-1 for 6 days, before plating for experiments as required.

#### Cell lines

THP1 cells were cultured in biotin-containing RPMI1640 medium with 2 mM L-glutamine, 10% FBS, 50 U/ml penicillin, 50 µg/ml streptomycin, 10 mM HEPES (Gibco) and 1 mM sodium pyruvate (Gibco). These cells were stimulated with 30 ng/ml PMA (Sigma-Aldrich) for 48 h, followed by PMA removal for 6 h before all functional experiments. PlatE, MEF, HEK293 (ATCC) and HEK293T cells were cultured in DMEM (Invitrogen) with 10% FBS, 50 U/ml penicillin and 50 µg/ml streptomycin. When plating for experiments, MEF cells were serum starved overnight (∼16 h) in the presence of DMEM (Invitrogen) with 0.5% FBS, 50 U/ml penicillin and 50 µg/ml streptomycin. The murine macrophage-like cell line RAW264.7 (ATCC) was cultured in RPMI1640 medium containing 2 mM Glutamax, 5% FBS, 50 U/ml penicillin and 50 µg/ml streptomycin. Further details of cell lines are in Table S2.

#### Ethics statement

All animal studies had approval from the appropriate University of Queensland Animal Ethics Committee. All work involving human primary cells was approved by the University of Queensland Institutional Human Research Ethics Committee. Human blood was collected from heathy donors following informed consent, and deidentified buffy coats were obtained from Australian Red Cross Lifeblood for this study.

### Method details

#### Generation of lentiviral constructs

TurboID-Empty vector was generated by inserting the TurboID gene block in the doxycycline-inducible lentiviral vector-pF_TRE3G_PGK_puro (Murphy et al., 2013; Moujalled et al., 2013). HDAC7-FLAG-TurboID plasmid was generated by using restriction enzymes to insert HDAC7-FLAG into the TurboID-Empty vector. NKG7-V5-TurboID was generated by using restriction enzymes to insert NKG7-V5 into the TurboID-Empty vector. All plasmids had their sequence verified prior to use.

#### Gene overexpression by lentiviral transduction in THP1 cells

For each transfection, 2×10^6^ HEK293T cells were plated and transfected with 0.8 µg/ml of lentiviral packaging and envelope plasmids including pCMV-dR8.2dvpr and pCMV-VSV-G (Addgene), and 1.2/ml µg of plasmid DNA along with 60 µl Lipofectamine 2000 (Invitrogen) in 1.5 ml OptiMEM medium (Invitrogen). At 6 h post-transfection, the medium was replaced with complete THP1 medium, and the cells were incubated for 24 h. The viral supernatants were collected and then filtered using PVDF syringe filters (0.45 mm; Millipore) and supplemented with 6 µl polybrene (Merck). This mix was then added to 2 wells of 2×10^6^ THP1 cells and spinfected at 1000 ***g*** at 35°C for 2 h. At 48 h post-transduction, the cells were washed with medium thrice and cultured in 1 µg/ml puromycin (Sigma-Aldrich)-containing medium. The differentiated cells were then stimulated with 100 ng/ml doxycycline (Sigma-Aldrich) to confirm inducible gene expression. For experiments, THP1 stable cell lines were differentiated with 30 ng/ml PMA for 48 h, after which cells were stimulated with or without doxycycline (100 ng/ml) for 16 h for proteomic studies or for 6 h for Class IIa HDAC enzyme activity assays, prior to subsequent analyses.

#### Construction of *Drp1*-deficient RAW264.7 cells

A combined protocol from Lipofectamine CRISPRMAX (Invitrogen) and Integrated DNA technologies (IDT) was used to construct *Drp1*-deficient RAW264.7 cell lines. Three distinct guide RNA (gRNA) duplexes (1 μM) were generated by annealing three different CRISPR RNAs (crRNAs) (IDT; sequence details provided in Table S2) with a trans-activating crRNA (tracrRNA). A mixture of three different gRNA duplexes were used for transfection to maximise the chances of gRNA-dependent *Drp1* targeting and subsequent Cas9-mediated deletion. 1×10^5^ RAW264.7 cells per 500 μl per well were plated in a 24-well plate on the day before transfection. A transfection mix was prepared containing 7 μl of the gRNA mix, 0.9 μl of Cas9 (8 μM), 0.25 μg blasticidin-resistant selection vector pEF6/V5-His TOPO (Thermo Fisher Scientific), 125 μl lipofectamine Cas9 plus (Invitrogen) and 31.25 μl OptiMEM medium. This was added to 31.25 μl of OptiMEM medium containing 1.9 μl CRISPRMAX Lipofectamine reagent (Invitrogen) and left at room temperature for 10 min, followed by dropwise addition to the previously plated RAW264.7 cells. The cells were incubated for 4 h at 37°C and then washed once before replenishing with fresh medium, supplemented with 2 μg/ml blasticidin. After overnight incubation and 24 h of transfection, the medium was replaced with fresh blasticidin-containing medium. After 48 h of selection, cells were transferred to a 10 cm tissue culture dish without blasticidin. The cells were then left undisturbed at 37°C until they formed visible colonies. Single colonies were then picked using pipette tips and transferred into individual wells of a 12-well plate. Once confluent, cells were split and again transferred to Sterilin plates. At this stage, whole cell lysates were harvested and screened for absence of DRP1 by western blotting.

#### Class IIa HDAC enzyme activity assay

1×10^5^ cells were plated in a black 96-well plate (Corning) and incubated at 37°C overnight. After appropriate treatments, cells were lysed in 10 µl mild lysis buffer (50 mM Tris-HCl, 150 mM NaCl, 1 mM EDTA, 1% NP40, pH 7.4) on ice for 15 min. Class IIa HDAC assay buffer (50 mM Tris-HCl, 137 mM NaCl, 2.7 mM KCl, 1 mM MgCl_2_, pH 8) containing 200 µM class IIa HDAC substrate [BOC-Lys(trifluoroacetyl)-AMC; Lobera et al., 2013] was added to the lysates and then incubated at 37°C for 30 min. After incubation, the reaction was stopped by adding 20 mM SAHA (Iyer et al., 2010; manufactured in-house) and 1 mg/ml trypsin (Invitrogen) diluted in class IIa HDAC assay buffer. The fluorescence of the resulting solution was measured using a plate reader (Infinite M Plex, Tecan) at the excitation wavelength and emission wavelength of 350 nm and 460 nm, respectively.

#### Streptavidin pulldown

The streptavidin pulldown protocol was modified from a previous study (Xiong et al., 2021). 30×10^6^ PMA-differentiated THP1 cells were washed using PBS and lysed using RIPA buffer (50 mM Tris-HCl, 150 mM NaCl, 0.1% SDS, 1% sodium deoxycholate, 1% NP40) supplemented with phosphatase inhibitor (PhosStop™, Roche) and protease inhibitor cocktail (cOmplete™, Roche). Next, 2 µl benzonase (Sigma-Aldrich) was added per sample and incubated for 20 min at room temperature to remove nucleic acids. The samples were then subjected to centrifugation at maximum speed (17,000 ***g***), and then a quarter of the lysate was stored as input. The lysates were passed through a PD-10 desalting column (GE Healthcare) to eliminate the presence of free biotin. Dynabeads MyOne streptavidin C1 (Thermo Fisher Scientific) were washed three times using RIPA buffer, and 400 µl of the beads was added to each sample. These samples were incubated overnight at 4°C. Following incubation, the samples were placed on a magnetic rack, and beads were washed twice with RIPA buffer, once in SDS wash buffer (2% SDS in 50 mM Tris-HCl pH 7) and once in urea buffer (2 M urea in 10 mM Tris-HCl pH 8). Finally, the beads were washed and resuspended in 50 µl 0.1 M ammonium bicarbonate for downstream applications.

#### Mass spectrometry

200 µl of sequencing grade Trypsin/Lys-C (Promega; 20 ng/µl in 50 mM ammonium bicarbonate pH 8) was added to each sample and incubated overnight at 37°C. After incubation, the beads were separated, and the sample was dried using vacuum centrifugation and reconstituted in 0.1% formic acid. The sample was analysed using liquid chromatography tandem mass spectrometry (LC-MS/MS) on a Triple TOF 6600 mass spectrometer (SCIEX).

#### Proteomic analysis

The initial mass spectrometry (MS) peak area analysis was performed using Skyline software (MacCoss Lab; https://skyline.ms/project/home/begin.view). Subsequently, protein and peptide identification from the final MS/MS spectra was performed using ProteinPilot™ software (SCIEX). The MS/MS spectra were mapped against Uniprot database (*Homo sapiens*), and the search parameter was set to conduct false discovery rate (FDR) analysis with a 1% global FDR cut-off for protein identification. The resulting data were then analysed to identify the unique and overlapping proteins in each sample. Pathway analysis was performed using the ClusterProfiler package in R (Yu et al., 2012). 104 HDAC7 candidate proteins were subjected to GO cellular component, biological process and molecular function analyses as well as KEGG pathway analysis in ClusterProfiler package in R. Furthermore, the interaction between glycolysis-related proteins was analysed using STRING (Szklarczyk et al., 2014).

#### Protein lysates and immunoblotting

Cells were lysed using RIPA buffer supplemented with phosphatase inhibitor (PhosStop™) and protease inhibitor cocktail (cOmplete™) and homogenised using a 21-gauge needle. 1× loading buffer (Bolt™ LDS sample buffer, Invitrogen) and 1× reducing agent (NuPAGE sample reducing agent, Invitrogen) was added to the lysates, which were then heat denatured at 100°C for 10 min. Samples were then loaded on 4–20% precast gels (Bio-Rad) and subjected to electrophoresis at 200 V for 30 min. The samples were transferred onto a nitrocellulose membrane (Bio-Rad) using a semi-dry Turbo-transfer system (Bio-Rad) at 25 V for 9 min. Membranes were blocked in 5% skimmed milk made in Tris-buffered saline (10 mM Tris-HCl, 150 mM NaCl) with 0.05% Tween-20 (TBST) for 1 h and then incubated with primary antibody (diluted in 5% milk or 5% BSA in TBST) overnight. The membranes were washed with TBST and incubated with secondary antibody (diluted in 5% milk or 5% BSA in TBST) along with Rhodamine-conjugated antibodies (against tubulin or GAPDH) for 1 h before being visualised using Clarity ECL (Bio-Rad) on the Bio-Rad Chemidoc-MP system (Bio-Rad). Further details of antibodies are in Table S2.

#### Mammalian expression constructs

The pEF6-HDAC7-V5, pEF6-HDAC7-ED-V5 (pEF6-HDAC7H649A-V5), pEF6-HDAC5-V5, pEF6-HDAC6-V5 and pEF6-SCIMP-V5 constructs have previously been described (Das Gupta et al., 2020; Luo et al., 2017; Shakespear et al., 2013; Taguchi et al., 2007). The pMIGR-HDAC7 and pMIGR-HDAC7-ED constructs used for retroviral-mediated gene expression have been previously described (Das Gupta et al., 2020). The full-length mouse DRP1 transcript variant 2 (mouse isoform b, NCBI Reference Sequence: NM_001025947.2) (without stop codon) was amplified by PCR from a pool of BMM cDNA (forward primer, 5′-GTCATGGAGGCGCTGATCC-3′; reverse primer, 5′-GAAATCCGAGAGACTCATCTTTGG-3′)*.* The amplified construct was then cloned into the pEF6/V5-His TOPO TA expression vector (Invitrogen) to generate the mouse pEF6-DRP1 construct. The DRP1 phosphomutant constructs, pEF6-DRP1-S635A and pEF6-DRP1-S656E (pEF6/V5-His TOPO TA backbone), were purchased from Gene Universal (USA). Endotoxin-free plasmid maxi-prep kits (Qiagen) were used to generate plasmid DNA for transfection.

#### Co-immunoprecipitation

For assessing interactions of endogenous proteins, 10×10^6^ THP1 cells were PMA-differentiated for 48 h, following which they were cross-linked with 1% formaldehyde (Thermo Fisher Scientific) for 10 min. Reactions were then quenched using 0.1 M glycine. Cells were washed twice with ice-cold PBS and then lysed in RIPA buffer containing protease inhibitor cocktail and phosphatase inhibitor. A tenth of the lysate volume was stored as input. For assessing interactions of ectopically expressed proteins, HEK293T cells were transfected with indicated constructs for 24 h, after which cells were lysed in RIPA buffer containing protease inhibitor cocktail and phosphatase inhibitor. 2 µl benzonase was added per sample and incubated for 20 min at room temperature to remove nucleic acids. The samples were then subjected to centrifugation at maximum speed (17,000 ***g***), and a quarter of the lysate was stored as input. 1 µg of mouse anti-DRP1 or mouse IgG1 isotype control antibody (see Table S2) was added to each sample and incubated overnight at 4°C. 25 µl Dynabeads protein G magnetic beads (Invitrogen) were then added to each sample and incubated on a rotor for 2 h at 4°C. The beads were then washed thrice with RIPA buffer and eluted using 1× loading buffer (Bolt™ LDS sample buffer, Invitrogen) and 1× reducing agent (NuPAGE sample reducing agent, Invitrogen). The samples were immunoblotted as described above.

#### Gene overexpression by retroviral transduction in BMMs

2×10^6^ PlatE cells per transfection were plated on 10 cm dishes and were transfected with 24 µg of Empty Vector plasmid DNA or molar equivalent of the indicated constructs along with 30 µl Lipofectamine 2000 in 1.5 ml OptiMEM medium. At 24 h post-transfection, the medium was replaced with complete BMM medium, and the cells were incubated at 32°C for another 48 h. The viral supernatants were collected, filtered using a PVDF syringe filter (0.45 µm) and supplemented with 1 M HEPES, 10 µg/ml polybrene and 150 ng/ml CSF1 and then incubated at room temperature for 20 min. The viral supernatants were then added to day 2 BMMs and spinfected at 1000 ***g*** at 35°C for 2 h. On day 6 of BMM differentiation, cells were harvested and plated for experiments.

#### Transient transfection of MEF cells

*Drp1*-deficient MEF cells have previously been described (Wakabayashi et al., 2009). 4.5×10^4^ WT or *Drp1*-deficient MEF cells were plated in a 24-well plate with serum-starved MEF media and incubated overnight at 37°C. On the following day, 0.5 μg of the indicated plasmids were transfected into MEF cells using Lipofectamine 3000 (Thermo Fisher Scientific), as per the manufacturer's protocol, after which the cells were incubated at 37°C. At 6 h post-transfection, the medium was replaced with complete MEF medium, with further analyses performed at 24 h post-transfection.

#### Proximity ligation assay

5×10^4^ BMMs were plated over the coverslips in a 24-well plate and fixed in 4% paraformaldehyde for 15 min. Cells were then washed three times with PBS and incubated with blocking buffer (1× PBS, 5% fetal calf serum, 0.3% Triton X-100) for 1 h. The blocking buffer was then replaced with the primary antibody diluted in the blocking buffer and kept at 4°C overnight. After washing with PBS, cells were incubated with Duolink In Situ Reagents Orange (Sigma) reagents according to the manufacturer's protocol. Cells were again washed with PBS and stained with wheat germ agglutinin–Alexa Fluor 647 (Invitrogen; 2.5 μg/ml) and 4′,6-diamidino-2-phenylidole (DAPI; Sigma-Aldrich; 20 ng/ml) for 20 min. Cells were washed three times with PBS, and the coverslips were mounted using IMBiol mounting media (Gelvatol mounting medium, IMB). Subsequently, slides were imaged using a Zeiss Axiovert 200 Inverted Microscope (Zeiss) with a 40× or 63× objective. Cells were defined using wheat germ agglutinin and DAPI, and the PLA puncta were identified using ImageJ software (National Institutes of Health, MD, USA). The threshold for the identification of puncta-positive cells was set to ≥3 puncta/cell. PLA-positive cells were counted for each sample, and the percentage of PLA-positive cells was plotted.

#### mRNA synthesis and Neon transfection of RAW264.7 cells

Mouse *Hdac7* and *Gfp* mRNA sequences were acquired from NCBI. Codon optimisation was performed using mRNAid (Vostrosablin et al., 2024), and the sequences were inserted between incomplete 5′ and 3′ UTRs designed for expression by the BASE Facility (The University of Queensland). DNA templates (synthesised by GenScript) were used in PCR reactions, from which mRNAs with complete UTRs were *in vitro* transcribed using the HiScribe™_T7 mRNA kit with CleanCap reagent AG (New England Bioscience), as per the manufacturer’s instructions, except standard UTP was substituted with 5-methoxy UTP (Thermo Fisher Scientific, Milwaukee) to reduce immunogenicity (Moradian et al., 2022). mRNA was purified using a Monarch RNA clean up kit (NEB), as per the manufacturer’s instructions, and transfected into RAW264.7 cells with the Neon NxT Electroporation System (100 µl kit, Thermo Fisher Scientific) using 1400 V, 20 ms and two pulses. Transfected cells were immediately transferred into antibiotic-free medium and plated as required for the different assays.

#### Real-time analysis of extracellular acidification rate

For real time analysis of ECAR, transfected RAW264.7 cells were plated at 5×10^4^ cells/ml in XF96 cell culture plates (Agilent) overnight. The following day, cells were washed and left for 1 h in unbuffered Seahorse XF DMEM medium (Agilent) supplemented with glucose (10 mM), glutamine (2 mM) and pyruvate (1 mM). ECAR measurements were performed using a Seahorse XFe96 Extracellular Flux Analyser (Agilent). Three baseline readings were taken before LPS (0.5 or 100 ng/ml) was added to the cells. Changes in ECAR were measured post-LPS stimulation for a period of up to 3 h. MTT assays (Sigma-Aldrich) were performed in parallel, and ECAR values were normalsed to MTT data to account for any variations in plating densities.

#### RNA extraction and qPCR

5×10^5^ cells were lysed in 350 µl of RLT buffer (Qiagen), after which total RNA was extracted using QIAGEN RNeasy extraction kits, as per manufacturer’s guidelines. cDNA was synthesised from 1 µg of RNA using oligo dT priming and Superscript III reverse transcriptase (Invitrogen), as per the manufacturer’s instructions. A no reverse transcription control was generated from RNA pooled from all samples in the preparation and treated as above but without the addition of reverse transcriptase. The synthesised cDNA was diluted 10-fold in ultrapure DNase/RNase-free water (Gibco) and used for quantitative PCR (qPCR) analysis. mRNA levels of *Ccl2*, *Edn1* and *Tnf*, relative to the housekeeping gene hypoxanthine phosphoribosyltransferase (*Hprt*), were determined by qPCR using the delta Ct method (Applied Biosystems; Livak and Schmittgen, 2001), SYBR Green PCR MasterMix (Applied BioSystems) and a Viia7 RT-PCR system (Applied BioSystems).

#### Mitotracker staining and quantification of mitochondrial numbers

1×10^5^ cells were plated over the coverslips in a 24-well plate and incubated at 37°C overnight. After the indicated treatments, the medium was replaced with fresh medium containing 150 nM (for macrophages) or 200 nM (for MEF cells) MitoTracker™ Deep Red FM (Invitrogen) for 20 min. Cells were then washed three times with PBS and fixed in 4% paraformaldehyde for 15 min. Cells were again washed three times with PBS and stained with 20 ng/ml DAPI for 20 min. After washing, the coverslips were mounted using IMBiol mounting medium and imaged using a Zeiss Axiovert 200 Inverted Microscope with 40× or 63× magnification objectives (mitochondria, red; nuclei, blue). For mitochondrial quantification, 15–30 cells were randomly selected from a minimum of five independent fields of view to quantify mitochondrial numbers by identifying maxima of intensity using ImageJ, as previously described (Afroz et al., 2022). Data from multiple experiments were combined for statistical analysis, and violin plots of representative experiments were used to show variability in mitochondria numbers across cell populations within each experiment. An open-source tool, Mitochondrial Analyzer, integrated as a plugin within ImageJ, was used to quantify mitochondrial branch length and form factor (https://github.com/AhsenChaudhry/Mitochondria-Analyzer; Chaudhry et al., 2020). Image thresholding was applied using a block size of 2.25 and C-value of 5 for all images. The mitochondrial network was analysed for each image using the default settings within the plugin.

### Quantification and statistical analysis

Statistical analysis was performed using Prism 10 (GraphPad). Data from three or more independent experiments were combined and displayed as mean with s.e.m., with different symbols used to indicate data from individual experiments. Figure legends show details of specific statistical analysis for different data sets, with *n* indicating number of independent experiments that were performed (either combined data where statistical analysis was performed or representative experiments for immunoblots and microscopy images).

## Supplementary Material

10.1242/joces.264376_sup1 Supplementary information

Table S1. List of 104 proteins that were specifically identified in streptavidin pulldowns for HDAC7-TurboID (versus NKG7-TurboID and empty vector-TurboID) in all three biological replicate experiments.
