# Supplementary material for: Histone deacetylase 7 mediates lipopolysaccharide-inducible mitochondrial fission in macrophages
Source: J Cell Sci. 2025 Oct 10;138(19):jcs264376. doi: 10.1242/jcs.264376 (PMC12539210; doi:10.1242/jcs.264376)
Supplement: Supplementary information [file joces-138-264376-s1.pdf]

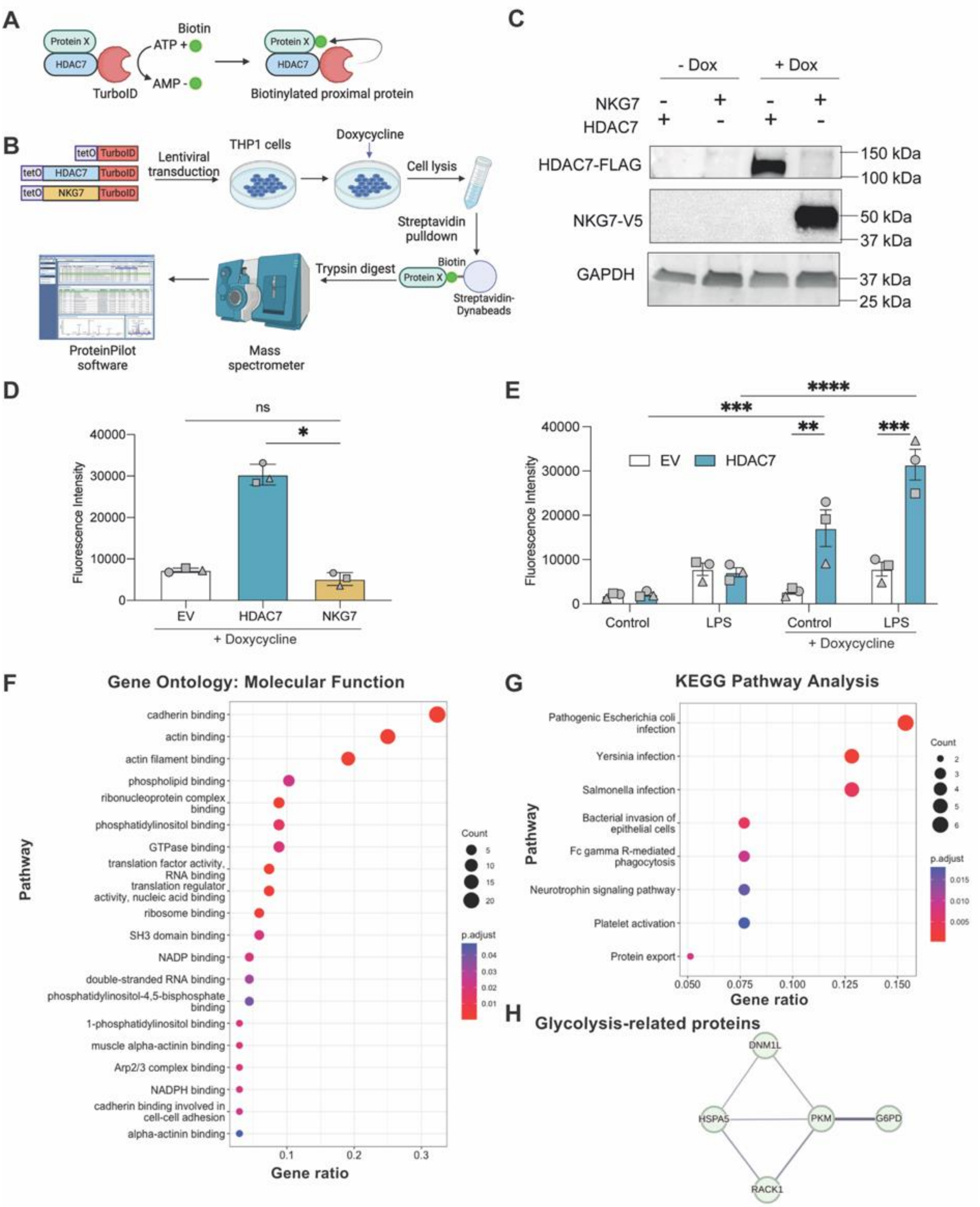

Fig. S1. Generation and functional validation of THP1 stable cell lines expressing TurboID fusion proteins.

(**A**) A schematic diagram of the TurboID system.

(**B**) Lentiviral vectors expressing HDAC7-FLAG-TurboID, NKG7-V5-TurboID fusion proteins or TurboID alone were transduced into THP1 cells. THP1 stable cell lines were

Journal of Cell Science • Supplementary information

differentiated with PMA for 48 h, then stimulated ± doxycycline (100 ng/mL) for 16 h. Cells were then lysed, and the biotinylated proteins were isolated using streptavidin pulldown. The samples were then digested using trypsin and proteins identified by mass spectrometry analysis. **(C)** Lentiviral vectors encoding HDAC7-FLAG-TurboID and NKG7-V5-TurboID fusion proteins were transduced in THP1 cells. Two sets of stable cell lines (generated in independent experiments) were selected using 1 µg/mL puromycin for a week, PMA-differentiated for 48 h, then stimulated ± doxycycline (100 ng/mL) for 16 h. Immunoblotting for epitope tags were used to visualise HDAC7 (anti-FLAG), NKG7 (anti-V5), and GAPDH as a loading control. Displayed immunoblots are representative of three independent experiments.

(**D**) Empty Vector (EV), HDAC7-TurboID, and NKG7-TurboID THP1 stable cell lines were PMA-differentiated for 48 h, then treated with doxycycline (100 ng/mL) for 16 h, after which cells were lysed, and class IIa HDAC enzyme activity was determined. Data are combined from three independent experiments (mean ± SEM, * p<0.05, RM one-way ANOVA followed by Dunnett's correction).

(**E**) Empty vector (EV) and HDAC7-TurboID THP1 stable cell lines were PMA-differentiated for 48 h, then stimulated ± doxycycline (100 ng/mL) for 6 h. Cells were then washed, left for a further 6 h in fresh media, then stimulated ± LPS (10 ng/mL) for 1 h. Cells were then lysed, and class IIa HDAC enzyme activity was determined. Data are combined from three independent experiments (mean ± SEM, ns – not significant, ** p<0.01; *** p<0.001, **** p<0.0001, RM two-way ANOVA followed by Sidak's multiple comparison test).

(**F-G**) The 104 proteins identified as being unique to the HDAC7-TurboID pulldown (candidate HDAC7 partners, **Figure 1**) were subjected to (**E**) Gene Ontology-Molecular Function and (**F**) KEGG Pathway enrichment analysis using the ClusterProfiler package. The dot plots above represent the pathways enriched in proteins identified from cells expressing HDAC7-FLAG-TurboID. The size of the dot indicates the protein count, and the colour indicates the p-values. *p*-values in **(G)** are nominal, without post-corrections, and only "*Pathogenic E. coli infection*" and "*Yersinia infection*" pathways remained significant post-FDR correction. Data are combined from three independent experiments.

(**H**) Protein-protein interaction network of the glycolysis-related proteins identified in the candidate HDAC7 partners. The protein interaction analysis was performed using STRING. A and B were created in BioRender by Abrol, R., 2025. https://BioRender.com/2d4wnk2. These panels were sublicensed under CC-BY 4.0 terms.

Journal of Cell Science • Supplementary information

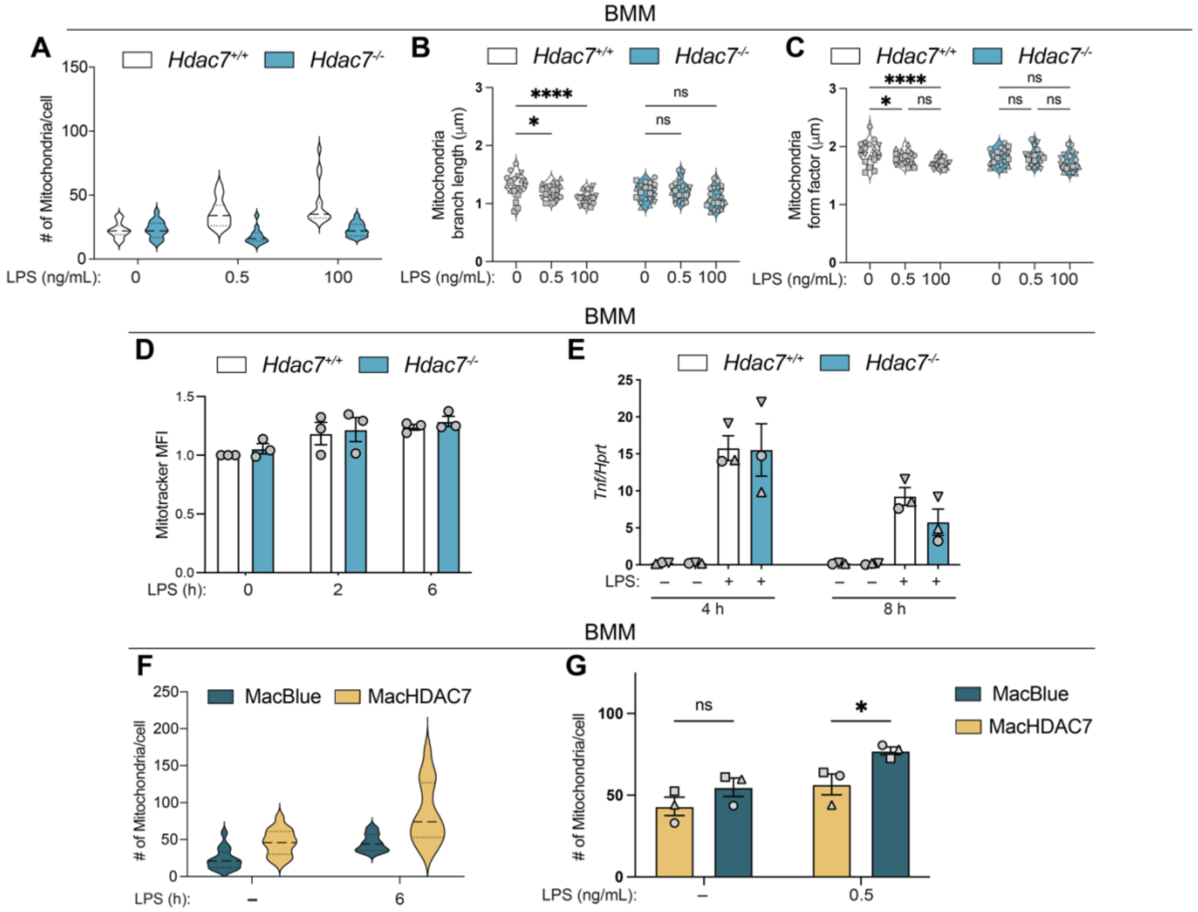

Fig. S2. Effects of HDAC7 on mitochondrial fission, mitochondrial mass, and inflammatory responses in macrophages.

(**A**) BMM from *Hdac7*$^{+/+}$ and *Hdac7*$^{-/-}$ mice were stimulated with LPS (0.5 ng/mL or 100 ng/mL) for 6 h. Cells were stained with 100 nM MitoTracker, then visualized by confocal microscopy. The numbers of mitochondria per cell from images were quantified by identifying maxima of intensity using ImageJ. 15 cells were counted per condition across five independent fields of view.

(**B-C**) BMM from *Hdac7*$^{+/+}$ and *Hdac7*$^{-/-}$ mice were stimulated with LPS (0.5 ng/mL or 100 ng/mL) for 6 h. Cells were stained with 100 nM MitoTracker and mitochondrial branch length (**B**) and form factor (**C**) were determined by Mitochondria Analyzer plugin in ImageJ (mean ± SEM, n=4, * $p< 0.05$, **** $p< 0.0001$, ns = not significant, RM two-way ANOVA, Sidak's multiple comparisons test).

(**D**) BMM from *Hdac7*$^{+/+}$ and *Hdac7*$^{-/-}$ mice were stimulated with LPS (100 ng/mL) for 2 or 6 h. Cells were stained with 100 nM MitoTracker and relative mitochondrial mass was determined by flow cytometry (mean ± SEM, data normalized to unstimulated *Hdac7*$^{+/+}$ controls, n=3).

Journal of Cell Science • Supplementary information

(**E**) BMM from *Hdac7*$^{+/+}$ and *Hdac7*$^{-/-}$ mice were stimulated with LPS (10 ng/mL) for 4 or 8 h, after which mRNA levels of *Tnf* (relative to *Hprt*) were quantified by qPCR (mean ± SEM, n=3).

(**F**) BMM from MacBlue and MacHDAC7 mice were stimulated with LPS (100 ng/mL) for 6 h. Cells were stained with 150 nM MitoTracker, then visualized by confocal microscopy. Numbers of mitochondria per cell were quantified by identifying maxima of intensity using ImageJ. 18 cells were counted per condition across five independent fields of view.

(**G**) BMM from MacBlue and MacHDAC7 mice were stimulated with 0.5 ng/mL LPS for 6 h. Cells were stained with 150 nM MitoTracker, then visualized by confocal microscopy. Numbers of mitochondria per cell were quantified by identifying maxima of intensity using ImageJ (mean ± SEM, * $p < 0.05$, RM two-way ANOVA, Sidak's multiple comparisons test, n=3).

Journal of Cell Science • Supplementary information

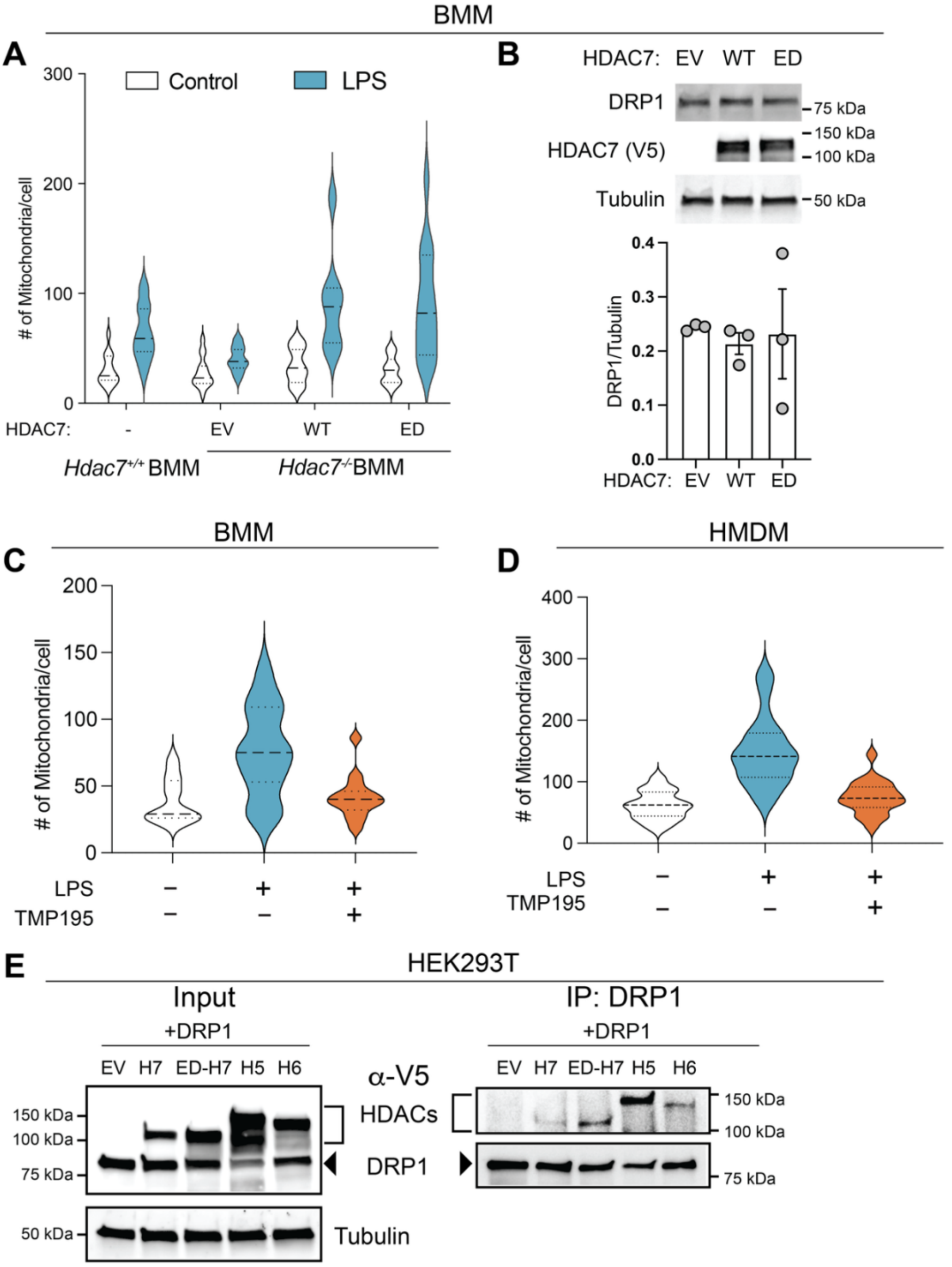

Fig. S3. Role of HDAC7 enzyme activity in LPS-inducible mitochondrial fission.

(**A**) BMM from myeloid *Hdac7*$^{-/-}$ mice were retrovirally transduced with empty vector (EV), a wild-type (WT) HDAC7 construct or an enzyme-dead HDAC7 mutant (ED) construct. BMM from wild-type C57BL/6 mice (WT BMM) were used as a control population. Cells from the indicated populations were stimulated with LPS (100 ng/mL) for 6 h and then stained with 150 nM MitoTracker (mitochondria in red) and 20 ng/mL DAPI (nuclei in blue) and visualized by confocal microscopy. Numbers of mitochondria per cell were

Journal of Cell Science • Supplementary information

quantified using ImageJ by identifying maxima of intensity. 20 cells were counted per condition across five independent fields of view.

(**B**) Retrovirally transduced BMM from myeloid *Hdac7*$^{-/-}$ mice were transduced with constructs as described in (**A**), after which DRP1, HDAC7 (V5) and Tubulin levels were assessed by immunoblotting. Representative immunoblots are displayed (top), with total DRP1 levels relative to Tubulin being quantified (bottom, mean ± SEM, n=3).

(**C**) BMM from C57BL/6 mice were pre-treated with either DMSO or TMP195 (0.5 μM) for 1 h and then stimulated with LPS (100 ng/mL) for 6 h. Cells were stained with 150 nM MitoTracker, then visualized by confocal microscopy. Numbers of mitochondria per cell were quantified using ImageJ by identifying maxima of intensity. 20 cells were counted per condition across five independent fields of view.

(**D**) HMDM were pre-treated with either DMSO or TMP195 (10 μM) for 1 h, then stimulated with LPS (100 ng/mL) for 6 h. Cells were stained with 150 nM MitoTracker, then visualized by confocal microscopy. The numbers of mitochondria per cell were quantified using ImageJ by identifying maxima of intensity. In the violin plots, the middle dark dotted line in the violin plot indicates median, while the light dotted line indicates the quartiles. A minimum of 15 cells were counted per condition across five independent fields of view.

(**E**) HEK293T cells were co-transfected with V5-tagged DRP1 and either empty vector (EV), HDAC7-V5 (H7), ED-HDAC7-V5 (ED-H7), HDAC5-V5 (H5) or HDAC6-V5 (H6). Cells were lysed, samples were immunoprecipitated (anti-DRP1), and immunoblotting was used to visualise the V5 tag (detecting both DRP1 and candidate interactors) and Tubulin (n=3).

Journal of Cell Science • Supplementary information

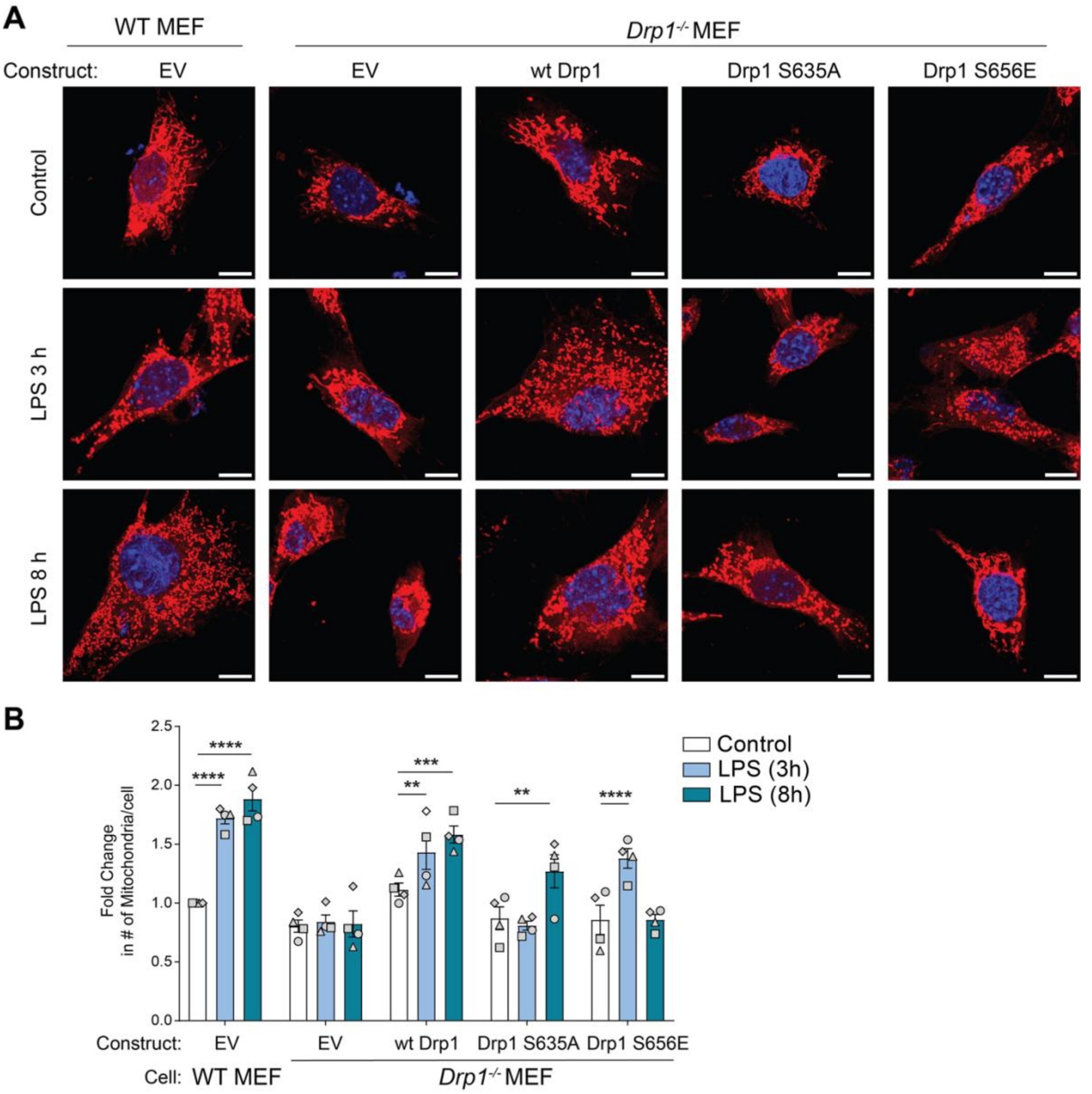

Fig. S4. Reconstitution of DRP1 in *Drp1*$^{-/-}$ MEF cells restores both stages of LPS-inducible mitochondrial fission.

(**A-B**) *Drp1*$^{-/-}$ MEF cells were transfected with an empty vector control (EV) or indicated DRP1 constructs, with EV-transfected wild type (WT) MEF cells included as a positive control. After 24 h, cells were stimulated with LPS (100 ng/mL) for 3 or 8 h, stained with MitoTracker (red) and DAPI (blue), visualized by confocal microscopy (**A**), and mitochondrial numbers per cell quantified (**B**) (scale = 10 µm, mean ± SEM, ** p< 0.01, *** p< 0.001, **** p< 0.0001, RM two-way ANOVA, Tukey's multiple comparisons test, n=4, data normalized to the unstimulated WT MEF-EV control).

Journal of Cell Science • Supplementary information

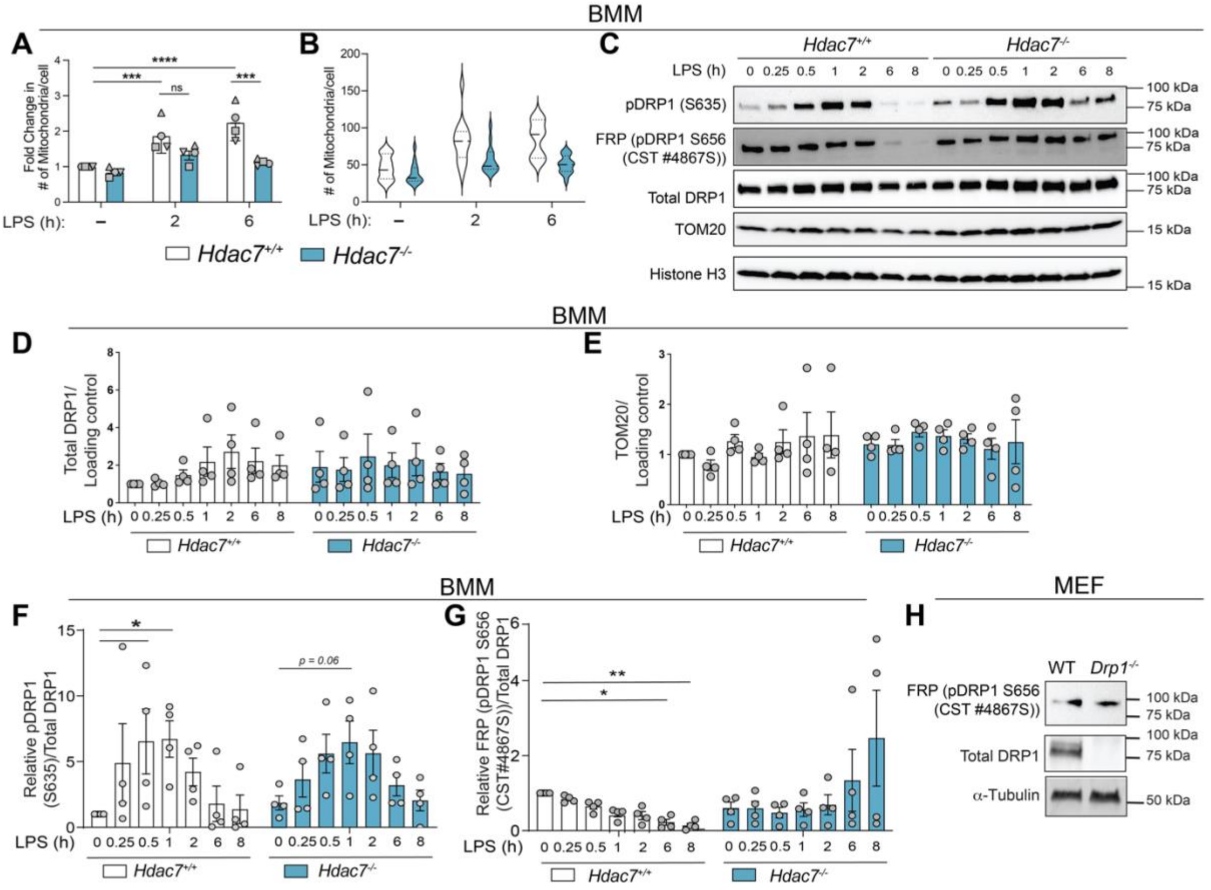

Fig. S5. Roles of HDAC7 in the two stages of LPS-inducible mitochondrial fission. (**A**) *Hdac7*$^{+/+}$ and *Hdac7*$^{-/-}$ BMM were stimulated with LPS (100 ng/mL) for 2 or 6 h. Cells were stained with MitoTracker (red) and DAPI (blue), visualized by confocal microscopy, and mitochondrial numbers per cell quantified (mean ± SEM, *** $p< 0.001$, **** $p< 0.0001$, RM two-way ANOVA, Dunnett's multiple comparisons test, n=4, normalized to the unstimulated control cells).

(**B**) A representative experiment from the compiled data in (**A**) showing numbers of mitochondria per cell, quantified by identifying maxima of intensity using ImageJ. In the violin plots, the middle dark dotted line in the violin plot indicates median, while the light dotted line indicates the quartiles. 20 cells were counted per condition across five independent fields of view.

(**C**) *Hdac7*$^{+/+}$ and *Hdac7*$^{-/-}$ BMM were stimulated with LPS (100 ng/mL), with whole cell extracts analysed by immunoblotting for pDRP1 (S635), FRP (detected by pDRP1 S656 antibody), total DRP1, TOM20 and Histone H3 (n=4).

(D-G) Quantification of data from (**C**) above. Intensity of total DRP1 relative to loading control (**D**), TOM20 relative to loading control (**E**), pDRP1 (S635) relative to total DRP1 levels

Journal of Cell Science • Supplementary information

(F) and FRP relative to total DRP1 levels (G) were quantified using Bio-Rad ImageLab software, with all data plotted relative to the *Hdac7*$^{+/+}$ unstimulated control cells (mean ± SEM, * $p< 0.05$, ** $p< 0.01$, RM two-way ANOVA, Dunnett's multiple comparisons test, n=4).

(H) Whole cell extracts from WT and *Drp1*$^{-/-}$ MEF cells were collected and analysed by western blot for FRP (detected by pDRP1 S656 antibody), total DRP1 and Tubulin (n=3).

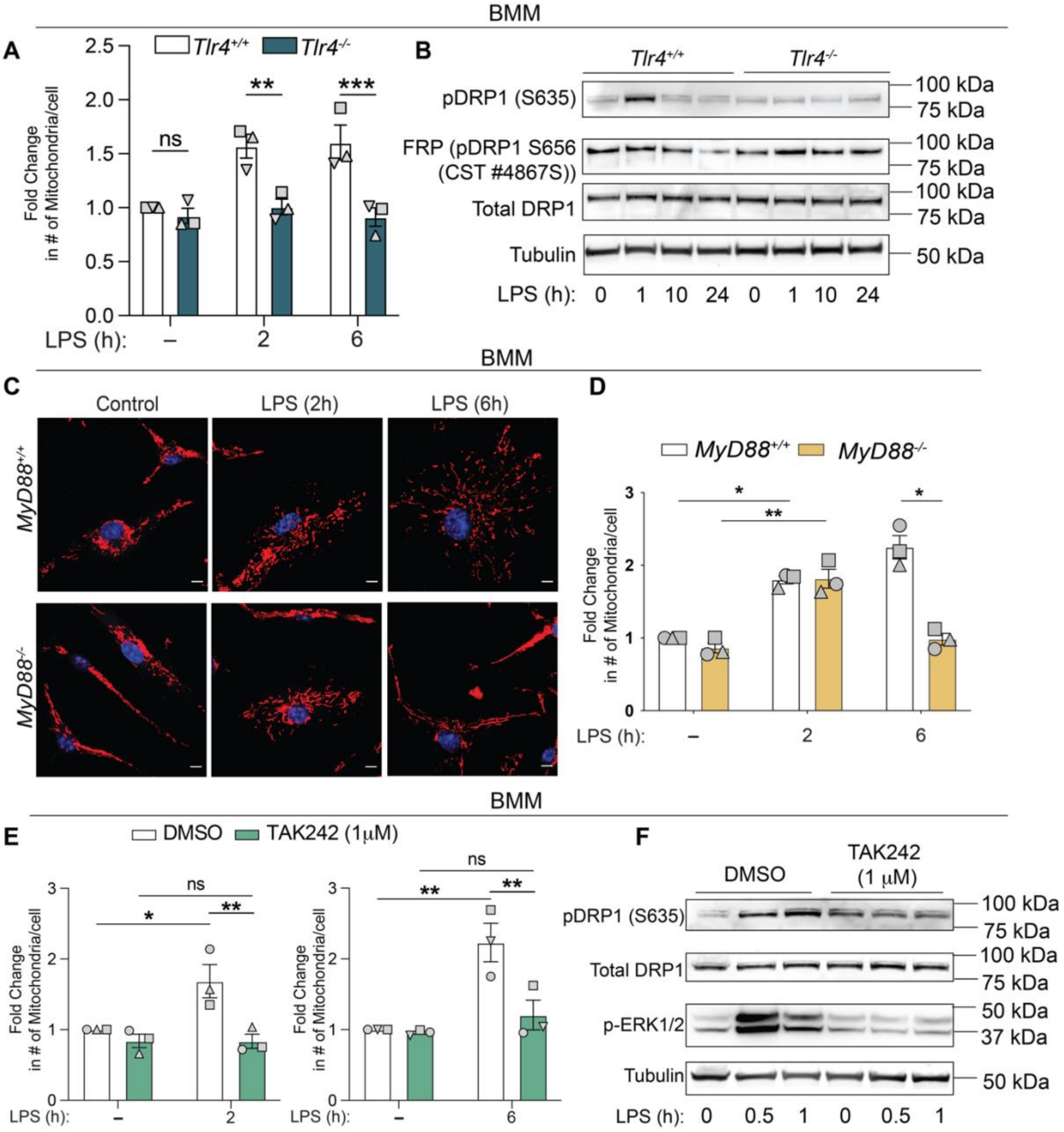

Fig. S6. TLR4 and TLR adaptors are essential for both stages of LPS-inducible fission in macrophages.

(**A**) BMM from *Tlr4*$^{+/+}$ and *Tlr4*$^{-/-}$ mice were stimulated with LPS (100 ng/mL) for 2 or 6 h. Cells were stained with 150 nM MitoTracker (mitochondria in red) and 20 ng/mL DAPI (nuclei in blue), then visualized by confocal microscopy. Numbers of mitochondria per cell were quantified by identifying maxima of intensity using ImageJ. Data (mean ± SEM, ** $p<0.01$, *** $p<0.001$, ns = not significant, RM two-way ANOVA, Sidak's multiple comparisons test, n=3, normalized to the *Tlr4*$^{+/+}$ unstimulated control cells).

(**B**) BMM from *Tlr4*$^{+/+}$ and *Tlr4*$^{-/-}$ mice were stimulated with LPS (100 ng/mL) for the indicated time points, after which whole cell extracts were collected and analysed by western blot for

Journal of Cell Science • Supplementary information

pDRP1 (S635), FRP (detected by pDRP1 S656 antibody), total DRP1 and Tubulin as a loading control (n=4).

(C) *MyD88*$^{+/+}$ and *MyD88*$^{-/-}$ BMM were stimulated with LPS (100 ng/mL) for 2 or 6 h. Cells were stained with MitoTracker (red) and DAPI (blue), then visualized by confocal microscopy (scale bar=5 μm).

(**D**) Numbers of mitochondria per cell in images from **C** were quantified (mean ± SEM, * p< 0.05, ** p< 0.01, RM two-way ANOVA, Sidak multiple comparisons test, n=3, normalized to the *MyD88*$^{+/+}$ unstimulated control cells).

(**E**) BMM from C57BL/6 mice were pre-treated with either DMSO or TAK242 (1 μM) for 1 h and then stimulated with LPS (100 ng/mL) for 2 or 6 h. Cells were stained with 150 nM MitoTracker and 20 ng/mL DAPI, then visualized by confocal microscopy. Numbers of mitochondria per cell were quantified using ImageJ by identifying maxima of intensity (mean ± SEM, * p<0.05, ** p<0.01, RM one-way ANOVA, Dunnett's multiple comparisons test, n=3, normalized to the unstimulated DMSO treated control).

(**F**) BMM from C57BL/6 mice were pre-treated with either DMSO or TAK242 (1 μM) for 1 h and then stimulated with LPS (100 ng/mL) for the indicated time points, after which whole cell extracts were collected and analysed by western blot for pDRP1 (S635), total DRP1, p-ERK1/2 (positive control), and Tubulin (n=3).

Table S1. List of 104 proteins that were specifically identified in streptavidin pulldowns for HDAC7-TurboID (versus NKG7-TurboID and empty vector-TurboID) in all three biological replicate experiments.

Available for download at
https://journals.biologists.com/jcs/article-lookup/doi/10.1242/jcs.264376#supplementary-data

Journal of Cell Science • Supplementary information

Table S2. Key resources table

REAGENT or RESOURCE	SOURCE	IDENTIFIER	
**Antibodies**			**Antibody conc. (dilutions)**
V5 (Mouse)	Bio-Rad	MCA1360	1 µg/mL (1:1000)
FLAG (Rabbit)	Sigma-Aldrich	F7425	0.8 µg/mL (1:1000)
DRP1 (Mouse)	Cell Signalling Technology	14647S	1.028 µg/mL (1:1000)
DRP1 (Rabbit)	Cell Signalling Technology	8570S	7.32 µg/mL (1:50)
HDAC7 (E708V) (Rabbit)	Cell Signalling Technology	10831	0.011 µg/mL (1:1000)
p38 MAPK (Rabbit)	Cell Signalling Technology	9212	0.068 µg/mL (1:1000)
phospho-DRP1 (S616) (Rabbit)	Cell Signalling Technology	3455S	0.12 µg/mL (1:1000)
phospho-DRP1 (S637) (Rabbit)	Cell Signalling Technology	4867S	0.067 µg/mL (1:1000)
phospho-ERK1/2 (Thr202/Tyr204) (Rabbit)	Cell Signalling Technology	9101S	0.191 µg/mL (1:1000)
Tom20 (Rabbit)	Cell Signalling Technology	42406S	0.051 µg/mL (1:1000)
Histone H3 (Mouse)	Cell Signalling Technology	14269	0.215 µg/mL (1:1000)
Mouse (MOPC-21) IgG1 Isotype Control	Cell Signalling Technology	4097S	
Tubulin (rhodamine conjugated)	Bio-Rad	12004165	N/A (1:10000)
GAPDH (rhodamine conjugated)	Bio-Rad	12004167	N/A (1:10000)
anti-mouse IgG HRP-linked antibody	Cell Signalling Technology	7076	0.061 µg/mL (1:2500)
anti-rabbit IgG HRP-linked antibody	Cell Signalling Technology	7074	0.026 µg/mL (1:2500)
**Chemicals, peptides, and recombinant proteins**			
lipopolysaccharide (LPS)	Sigma-Aldrich	L2137	
PMA	Sigma-Aldrich	P1585	

Journal of Cell Science • Supplementary information

lipofectamine 2000	Thermo Fisher Scientific	11668019	
lipofectamine 3000	Thermo Fisher Scientific	L3000015	
polybrene	Merck	TR-1003-G	
TMP195	Fairlie Laboratory, IMB	N/A	
puromycin	Sigma-Aldrich	P7255	
doxycycline	Sigma-Aldrich	D 9891	
cOmplete Protease inhibitor cocktail	Roche	11697498001	
PhosStop (phosphatase inhibitor)	Sigma-Aldrich	4906845001	
trypsin/Lys-C	Promega	V5073	
trypsin	Invitrogen	15090046	
SAHA	Fairlie Laboratory, IMB	N/A	
MitoTracker^{TM} Deep Red FM	Invitrogen	M22426	
wheat germ agglutinin	Life Technologies	W32466	
4',6-diamidino-2-phenylidole (DAPI)	Sigma-Aldrich	D9542	
Class IIa HDAC substrate (BOC-Lys(trifluoroacetyl)-AMC)	Fairlie Laboratory, IMB	N/A	
**Critical commercial assays**			
Duolink In Situ Reagents Orange	Sigma-Aldrich	DUO92007	
Duolink In Situ Probe Anti-Mouse Plus	Sigma-Aldrich	DUO92001	
Duolink In Situ Probe Anti-Rabbit Plus	Sigma-Aldrich	DUO92005	
Qiagen Endofree Plasmid Maxi Kit	Qiagen	12362	
**Experimental models: Cell lines**			
HEK293T	ATCC	CRL-3216	
PlatE	CELL BIOLABS, INC	RV-101	
THP1	ATCC	TIB-202	
WT MEF	Prof Mike Ryan (Monash Biomedicine Discovery Institute, Australia)	N/A	
Drp1$^{-/-}$ MEF	Prof Mike Ryan (Monash Biomedicine Discovery Institute, Australia)	N/A	
WT RAW264.7	ATCC	TIB-71	
Δ*Drp1* RAW264.7	This paper	N/A	
**Experimental models: Organisms/strains**			

Journal of Cell Science • Supplementary information

C57BL/6J	In-house breeding	N/A	
MacBlue	In-house breeding	N/A	
MacHDAC7	In-house breeding	N/A	
Hdac7$^{+/+}$*(Hdac7*$^{fl/fl}$*)*	Prof Eric Olson and Dr Rhonda Bassel-Duby (UT Southwestern Medical Centre, United States)	N/A	
Hdac7$^{-/-}$*(Hdac7*$^{fl/fl}$*/Lysm*^{Cre}*)*	In-house breeding	N/A	
Tlr4$^{+/+}$	In-house breeding	N/A	
Tlr4$^{-/-}$	In-house breeding	N/A	
MyD88$^{+/+}$	Blumenthal laboratory, TRI	N/A	
MyD88$^{-/-}$	Blumenthal laboratory, TRI	N/A	
**Oligonucleotides**			
DRP1 cloning primer<br><br>Fwd: GTCATGGAGGCGCTGATCC	This paper	N/A	
DRP1 cloning primer<br><br>Rev: GAAATCCGAGAGACTCATCTTTGG	This paper	N/A	
mRNA T7 complete primer Fwd: TAATACGACTCACTATAAGGACTCTTCTGGTCCCCACA	This paper	N/A	
mRNA PolyA complete primer Rev: T_{125}GCTAGCTCCAGGGTGTGGC	This paper	N/A	
Ccl2 qPCR primer<br><br>Fwd: GCTTCTTTGGGACACCTGCTG	Sweet Laboratory, IMB	N/A	
Ccl2 qPCR primer<br><br>Rev: CCCACTCACCTGCTGCTACTCA	Sweet Laboratory, IMB	N/A	
Edn1 qPCR primer<br><br>Fwd: GGAAGGAAGGAAACTACGAAGG	Sweet Laboratory, IMB	N/A	
Edn1 qPCR primer<br><br>Rev: GTGCGTCAACTTCTGGTCTCT	Sweet Laboratory, IMB	N/A	
Tnf qPCR primer<br><br>Fwd: CATCTTCTCAAAATTCGAGTGACAA	Sweet Laboratory, IMB	N/A	
Tnf qPCR primer<br><br>Rev: TGGGAGTAGACAAGGTACAACCC	Sweet Laboratory, IMB	N/A	
Hprt qPCR primer	Sweet Laboratory, IMB	N/A	

Journal of Cell Science • Supplementary information

Fwd: GCAGTACAGCCCCAAAATGG			
Hprt qPCR primer<br><br>Rev: AACAAAGTCTGGCCTGTATCCAA	Sweet Laboratory, IMB	N/A	
**crRNAs**			
GACACTTGTGGATTTACCGG	This paper	N/A	
GAGGAACTGGTGTGGTCACC	This paper	N/A	
CAGGACGTCTTCAACACAGT	This paper	N/A	
**Recombinant DNA**			
Lenti-TurboID-Empty vector	Sweet Laboratory, IMB	N/A	
Lenti-HDAC7-FLAG-TurboID	Sweet Laboratory, IMB	N/A	
Lenti-NKG7-V5-TurboID	Sweet Laboratory, IMB	N/A	
pCMV-dR8.2dvpr	Addgene	N/A	
pCMV-VSV-G	Addgene	N/A	
pEF6-DRP1-V5	Sweet Laboratory, IMB	N/A	
pEF6-DRP1-S635A	Sweet Laboratory, IMB	N/A	
pEF6-DRP1-S656E	Sweet Laboratory, IMB	N/A	
pEF6-HDAC7-V5	Sweet Laboratory, IMB	N/A	
pEF6-HDAC7-ED-V5	Sweet Laboratory, IMB	N/A	
pEF6-SCIMP-V5	Sweet Laboratory, IMB	N/A	
pEF6-HDAC5-V5	Sweet Laboratory, IMB	N/A	
pEF6-HDAC6-V5	Sweet Laboratory, IMB	N/A	
pMIGR-HDAC7-V5	Sweet Laboratory, IMB	N/A	
pMIGR-HDAC7-ED-V5	Sweet Laboratory, IMB	N/A	
**Software**			
ImageJ	FIJI	N/A	
Image Lab	Bio-Rad	N/A	
Prism 10	GraphPad	N/A	
ProteinPilot^{TM}	SCIEX	N/A	
Skyline	MacCoss Lab	N/A	
R package-clusterProfiler	Bioconductor	N/A	
